# Genome-Wide Identification of Susceptibility Alleles for Viral Infections through a Population Genetics Approach

**DOI:** 10.1371/journal.pgen.1000849

**Published:** 2010-02-19

**Authors:** Matteo Fumagalli, Uberto Pozzoli, Rachele Cagliani, Giacomo P. Comi, Nereo Bresolin, Mario Clerici, Manuela Sironi

**Affiliations:** 1 Scientific Institute IRCCS E. Medea, Bioinformatic Lab, Bosisio Parini (LC), Italy; 2 Bioengineering Department, Politecnico di Milano, Milan, Italy; 3 Dino Ferrari Centre, Department of Neurological Sciences, University of Milan, IRCCS Ospedale Maggiore Policlinico, Mangiagalli and Regina Elena Foundation, Milan, Italy; 4 Department of Biomedical sciences and Technologies LITA Segrate, University of Milan, Milan, Italy; 5 Don C. Gnocchi ONLUS Foundation IRCCS, Milan, Italy; Fred Hutchinson Cancer Research Center, United States of America

## Abstract

Viruses have exerted a constant and potent selective pressure on human genes throughout evolution. We utilized the marks left by selection on allele frequency to identify viral infection-associated allelic variants. Virus diversity (the number of different viruses in a geographic region) was used to measure virus-driven selective pressure. Results showed an excess of variants correlated with virus diversity in genes involved in immune response and in the biosynthesis of glycan structures functioning as viral receptors; a significantly higher than expected number of variants was also seen in genes encoding proteins that directly interact with viral components. Genome-wide analyses identified 441 variants significantly associated with virus-diversity; these are more frequently located within gene regions than expected, and they map to 139 human genes. Analysis of functional relationships among genes subjected to virus-driven selective pressure identified a complex network enriched in viral products-interacting proteins. The novel approach to the study of infectious disease epidemiology presented herein may represent an alternative to classic genome-wide association studies and provides a large set of candidate susceptibility variants for viral infections.

## Author Summary

Viruses have represented a constant threat to human communities throughout their history, therefore, human genes involved in anti-viral response can be thought of as targets of virus-driven selective pressure. Here we utilized the marks left by selection to identify viral infection-associated allelic variants. We analyzed more than 660,000 single nucleotide polymorphisms (SNPs) genotyped in 52 human populations, and we used virus diversity (the number of different viruses in a geographic region) to measure virus-driven selective pressure. Results showed that genes involved in immune response and in the biosynthesis of glycan structures functioning as viral receptors display more variants associated with virus diversity than expected by chance. The same holds true for genes encoding proteins that directly interact with viral components. Genome-wide analysis identified 441 variants, mapping to 139 human genes, significantly associated with virus-diversity. We analyzed the functional relationships among genes subjected to virus-driven selective pressure and identified a complex interaction network enriched in viral products-interacting proteins. Therefore, we describe a novel approach for the identification of gene variants that may be involved in the susceptibility to viral infections.

## Introduction

Infectious diseases represent one of the major threats to human populations, are still the first cause of death in developing countries [1], and are therefore a powerful selective force. In particular, viruses have affected humans before they emerged as a species, as testified by the fact that roughly 8% of the human genome is represented by recognizable endogenous retroviruses [2] which represent the fossil remnants of past infections. Also, viruses have probably acted as a formidable challenge to our immune system due to their fast evolutionary rates [3]. Indeed, higher eukaryotes have evolved mechanisms to sense and oppose viral infections; the recent identification of the antiviral activity of particular proteins such as APOBEC, tetherin, and TRIM5 has shed light on some of these mechanisms. Genes involved in anti-viral response have therefore been presumably subjected to an enormous, continuous selective pressure.

Despite the relevance of viral infection for human health, only few genome-wide association studies (GWAS) have been performed in the attempt to identify variants associated with increased susceptibility to infection or faster disease progression [4]–[5]. These studies have shown the presence of a small number of variants, mostly located in the HLA region. This possibly reflects the low power of GWAS to identify variants with a small effect. An alternative approach to discover variants that modulate susceptibility to viral infection is based on the identification of SNPs subjected to virus-driven selective pressure. Indeed, even a small fitness advantage can, on an evolutionary timescale, leave a signature on the allele frequency spectrum and allow identification of candidate polymorphisms. To this aim we exploited the availability of more than 660,000 SNPs genotyped in 52 human populations distributed world-wide (HGDP-CEPH panel) [6] and of epidemiological data stored in the Gideon database.

## Results

### Virus diversity is a reliable estimator of virus-driven selective pressure

Previous studies [7]–[9] have suggested that the number of the different pathogen species transmitted in a given geographic location is a good estimate of pathogen-driven selection for populations living in that area. Indeed, pathogen diversity is largely dependent on climatic factors [10] and might more closely reflect historical pressures than other estimates such as the prevalence of specific infections. We therefore reasoned that virus diversity can be used as a measure of the selective pressure exerted by virus-borne diseases on human populations and, as a consequence, that SNPs showing an unusually strong correlation with virus diversity can be considered genetic modulators of infection susceptibility or progression. To explore this possibility we used a large set of SNPs that have been genotyped in the HGDP-CEPH panel, a collection of DNAs from almost 950 individuals sampled throughout the world (Table 1). Virus diversity estimates were derived from the Global Infectious Disease and Epidemiology Network database: for each country where HGDP-CEPH populations are located we counted the number of different virus species (or genera/family as described in materials and methods) that are naturally transmitted (Table 1).

**Table 1:** Populations in the HGDP-CEPH panel and virus diversity estimates.

Population	Country	Sampled individuals	Virus diversity
Bantu North East	Kenya	11	49
Bantu South East	South Africa	8	46
Biaka Pygmies	Central African Republic	23	54
Mandenka	Senegal	22	51
Mbuti Pygmies	Democratic Republic of Congo	13	50
San	Namibia	5	42
Yoruba	Nigeria	21	54
Colombians	Colombia	7	49
Karitiana	Brazil	14	55
Maya	Mexico	21	49
Pima	Mexico	14	49
Surui	Brazil	8	55
Balochi	Pakistan	24	45
Brahui	Pakistan	25	45
Burusho	Pakistan	25	45
Hazara	Pakistan	22	45
Kalash	Pakistan	23	45
Makrani	Pakistan	25	45
Pathan	Pakistan	23	45
Sindhi	Pakistan	24	45
Uygur	China	10	47
Cambodians	Cambodia	10	42
Dai	China	10	47
Daur	China	9	47
Han	China	44	47
Hezhen	China	9	47
Japanese	Japan	29	41
Lahu	China	8	47
Miaozu	China	10	47
Mongola	China	10	47
Naxi	China	8	47
Oroqen	China	9	47
She	China	10	47
Tu	China	10	47
Tujia	China	10	47
Xibo	China	9	47
Yakut	Russia	25	48
Yizu	China	10	47
Adygei	Russia	17	48
French	France	28	42
French Basque	France	24	42
North Italian	Italy	13	43
Orcadian	Orkney Islands (Scotland)	15	39
Russian	Russia	25	48
Sardinian	Italy	28	43
Tuscan	Italy	8	43
Bedouin	Israel	46	41
Druze	Israel	42	41
Mozabite	Algeria	29	39
Palestinian	Israel	46	41
NAN Melanesian	Papua New Guinea	11	45
Papuan	Papua New Guinea	17	45

One simple prediction of our hypothesis whereby virus diversity is a reliable estimator of virus-driven selective pressure is that genes known to be involved in immune response are enriched in SNPs significantly associated with virus richness. In order to verify whether this is the case we analysed the InnateDB gene list which contains 2,915 genes involved in immune response and showing the presence of at least one SNP in the HGDP-CEPH panel. Correlations with virus richness were calculated using Kendall's partial rank correlation; since allele frequency spectra in human populations are known to be affected by demographic factors in addition to selective forces [11]–[12], each SNP was assigned a percentile rank in the distribution of τ values calculated for all SNPs having a minor allele frequency (MAF) similar (in the 1% range) to that of the SNP being analysed. A SNP was considered to be significantly associated with virus diversity if it displayed a significant correlation (after Bonferroni correction with α = 0.01) and a rank higher than 0.99. As shown in Table 2, 104 SNPs in InnateDB genes showed a significant association with virus diversity. All SNPs in InnateDB genes that correlated with virus diversity are listed in Table S1. By performing 10,000 re-samplings of 2,915 randomly selected human genes (see materials and methods for details) we verified that the empirical probability of obtaining 104 significantly associated SNPs amounts to 0.010, indicating that genes in the InnateDB list display more virus-associated SNPs than expected.

**Table 2:** Enrichment of SNPs significantly associated with virus diversity in different gene lists.

Gene list	Genes	SNPs	Corr. SNPs^a^	*p* value^b^	Contributing genes^c^
InnateDB	2915	59783	104	0.0105	*TNFRSF1B, HSPG2, KIAA0319L, PSMB2, NEGR1, CHIA, ARHGEF11, FCRLA, DDR2, HMCN1, IL19, LAMB3, TGFB2PRKCE, CLEC4F, POLR1A, LRP1B, LRP2, HDAC4, CNTN4, CLDN18, LPP, MAEA, C1QTNF7, PPP3CA, DCHS2, SEMA5A, PDZD2, SQSTM1, GMDS, GPLD1, CCND3, LAMA4, MMD2, CNTNAP2, TNFRSF10C, FREM1, COL5A1, NELL1, SERPING1, CTNND1, FCHSD2, CCND2, SCNN1A, ST8SIA1, PPFIBP1, PKP2, LIN7A, UNG, GALNTL1, BDKRB2, AQP9, IL16, CDH13, CBFA2T3, CDH15, SLFN5, DCC, FXYD5, CLDN14, DSCAM, ADARB1, TOM1, PARVG, CLDN2*
Glycan biosynthesis	200	5343	50	0.0138	*ST3GAL3, ST6GALNAC3, GALNT14, GALNT13, ST6GAL1, GALNT10, UST, WBSCR17, GALNTL5, ST3GAL1, UGCG, GALNTL4, B4GALNT3, ST8SIA1, GALNT6, GALNTL1, XYLT1, CHST6, HS3ST3A1, FUT6, LARGE, ST8SIA6, CHSY3, MGAT5B, TUSC3*
Host-virus interaction	1916	14746	80	0.0172	*ENO1, CAPZB, SFRS4, SFPQ, PDE4B, MSH2, PCAF, TMEM110, GTF2E1, ADCY5, PLS1, NUP43, AKAP12, RPA3, PDE1C, ABP1, MTDH, EIF3S3, SNTB1, PCSK5, GSN, VAV2, POLR3A, PDE2A, CENTD2, RPS3, GRIN2B, PTPRO, PDE3A, ITPR2, NR4A1, POMP, RFC3, PCCA, SIPA1L1, SPTBN5, PLA2G4F, CAPN3, GTF3C1, KARS, NF1, MGAT5B, GAA, IL4I1, VPS16, PTPRA, PLCB4, SREBF2*

**^a^**Number of SNPs showing significant correlation with virus diversity.

**^b^**The empirical *p* value was calculated as described in the text and in Materials and Methods.

**^c^**Genes showing at least one SNP significantly correlated with virus diversity.

It is worth mentioning that amongst these genes, *UNG* (MIM 191525), encoding uracil DNA glycosylase, functions downstream of *APOBEC3G* (MIM 607113) to mediate the degradation of nascent HIV-1 DNA [13]. *SERPING1* (MIM 606860), a regulator of the complement cascade, is also involved in HIV-1 infection (MIM 609423) as its expression is dysregulated in immature dendritic cells by Tat [14]; moreover, the protein product of *SERPING1* is cleaved by HCV and HIV-1 proteases [15]–[16].

Genes involved in the biosynthesis of glycan structures have also been considered as possible modulators of infection susceptibility. Indeed, since Haldane's prediction in 1949 [17] that antigens constituted of protein-carbohydrates molecules modulate the resistance/susceptibility to pathogen infection, protein glycolsylation has been shown to play a pivotal role in viral recognition of host targets [18], as well as in antigen uptake and processing and in immune modulation [19]–[20]. We therefore computed a list of genes involved in glycan biosynthesis from KEGG pathways and Gene Ontology annotations. Again these genes displayed significantly more virus-associated SNPs than expected if randomness alone were responsible (empirical *p* = 0.0138) (Table 2 and Table S2). Several virus-associated SNPs were located in genes coding for sialyltransferases (*ST6GAL1* (MIM 109675), *ST3GAL3* (MIM 606494), *ST6GALNAC3* (MIM 610133), *ST8SIA1* (MIM 601123), *ST3GAL1* (MIM 607187) and *ST8SIA6* (MIM 610139)). Notably, sialic acids represent the most prevalent terminal monosaccharides on the surface of human cells and determine the host range of different viruses including influenza A [21]–[22], polyomaviruses (i.e JCV and BKV in humans) [23], and rotaviruses (the leading cause of childhood diarrhea) [24].

Sialyltransferases also play central roles in B and T cell communication and function. In particular, the generation of influenza-specific humoral responses is impaired in mice lacking *ST6GAL1* [25], while *ST3GAL1* regulates apoptosis of CD8+ T cells [20]. Interestingly, *ST8SIA6* is expressed in NK cells, possibly playing a role in the regulation of Siglec-7 lectin inhibitory function in these cells [26]. Four other genes (*XYLT1* (MIM 608124), *HS3ST3A1* (MIM 604057), *UST* (MIM 610752) and *CHSY3* (MIM 609963)) carrying SNPs associated with virus diversity are involved in the biosynthesis of either heparan sulphate or chondroitin sulphate. The former is an ubiquitously expressed glycosaminoglycan serving as the cell entry route for herpesviruses [27], HTLV-1 [28] and papillomaviruses [29]. Chondroitin sulphate is similarly expressed on a wide array of cell types and functions as an auxiliary receptor for binding of herpes simplex virus [30] as well as a facilitator of HIV-1 entry into brain microvascular endothelial cells [31]. Finally, we identified *LARGE* (MIM 603590) among the genes subjected to virus-driven selective pressure (Table 2). Recent studies have demonstrated that the post-translational modification of α-dystroglycan by LARGE is critical for the binding of arenaviruses of different phylogenetic origin including Lassa fever virus and lymphocytic-choriomeningitis virus [32]–[33]. Therefore our data support the previously proposed hypothesis whereby viruses represent the selective pressure underlying the strong signal of positive selection at the *LARGE* locus [34].

Since genes involved in immune response and in the biosynthesis of glycan structures are likely to be subjected to selective pressures exerted by pathogens other than viruses, we verified whether a set of genes directly involved in interaction with viral proteins also displays more SNPs significantly correlated with virus diversity. To this aim we retrieved a list of 1,916 genes known to interact with at least one viral product and displaying at least one genotyped SNP in the HGDP-CEPH panel (see materials and methods). In order to perform a non-redundant analysis, genes included in the InnateDB list and involved in glycan biosynthesis were removed; the remaining 987 genes displayed 80 SNPs correlated with virus diversity, corresponding to an empirical *p* value of 0.017 (Table 2 and Table S3). Notably, when this same analysis was performed using the diversity of pathogens other than viruses (bacteria, protozoa and helminths), no significant excess of correlated SNPs was found (all empirical *p* values>0.05).

### Genome-wide identification of variants subjected to virus-driven selective pressure

Given these results, we wished to identify SNPs significantly associated with virus richness on a genome-wide base. We therefore calculated Kendall's rank correlations between allele frequency and virus diversity for all the SNPs (n = 660,832) typed in the HGDP-CEPH panel. We next searched for instances which withstood Bonferroni correction (with α = 0.05) and displayed a τ percentile rank higher than the 99^th^ among MAF-matched SNPs. A total of 441 SNPs mapping to 139 distinct genes satisfied both requirements. Table 3 shows the 30 top SNPs (or SNP clusters) located within genic regions and associated with virus diversity, while the full list of SNPs subjected to virus-driven selective pressure is available on Table S4. It is worth noting that the SNP dataset we used contains less than 200 variants mapping to *HLA* genes (both class I and II), therefore covering a minor fraction of genetic variability at these loci; as a consequence *HLA* genes cannot be expected to be identified as targets of virus-driven selective pressure using the approach we describe herein.

We next verified whether the correlations detected between the SNPs we identified and virus diversity could be secondary to climatic variables. Hence, for all countries where HGDP-CEPH populations are located we obtained (see materials and methods) the following parameters: average annual minimum and maximum temperature, and short wave (UV) radiation flux. Results showed that none of the SNPs associated with virus diversity significantly correlated with any of these variables (Table S5).

**Table 3:** Top 30 SNPs (or SNP clusters) correlated with virus diversity.

SNP	Gene symbol	Description	Annotation^a^	τ
rs10511316	*CCDC80*	coiled-coil domain containing 80	intron	0.627
rs1135029; rs189332; rs11235559	*PDE2A*	phosphodiesterase 2A, cGMP-stimulated	A867A; intron; intron	0.615
rs1011051; rs2278295	*MYO5C*	myosin VC	intron; intron	0.609
rs993715; rs2189883	*CNTNAP2*	contactin associated protein-like 2	intron; intron	0.609
rs11581	*KIAA1529*	-	Q1642Q	0.607
rs3785415	*CDH15*	cadherin 15, type 1, M-cadherin	intron	0.603
rs17256082	*SCRN3*	secernin 3	intron	0.600
rs4852988	*ANXA4*	annexin A4	intron	0.597
rs4575989; rs4629443	*C1QTNF7*	C1q and tumor necrosis factor related protein 7	intron; intron	0.597
rs7637370	*CLDN18*	claudin 18	intron	0.596
rs519332	*EYA4*	eyes absent 4 homolog	intron	0.596
rs2188172; rs11760238	*LHFPL3*	lipoma HMGIC fusion partner-like 3	intron; intron	0.595
rs1650893	*LOC51149*	-	Q42R	0.594
rs1322633	*RNF217*	ring finger protein 217	intron	0.593
rs7927476	*NELL1*	NEL-like 1	intron	0.593
rs2615666	*TMEM132B*	transmembrane protein 132B	intron	0.593
rs13020779	*DIS3L2*	DIS3 mitotic control homolog (S. cerevisiae)-like 2	intron	0.589
rs1719596	*LEPREL1*	leprecan-like 1	intron	0.589
rs1065154	*SQSTM1*	sequestosome 1	3′ UTR	0.589
rs12145973	*IL19*	Interleukin 19	intron	0.589
rs1890139	*PCCA*	propionyl Coenzyme A carboxylase, alpha polypeptide	intron	0.588
rs6505045	*ANKFN1*	ankyrin-repeat and fibronectin type III domain containing 1	intron	0.587
rs4953260	*PRKCE*	protein kinase C, epsilon	intron	0.587
rs4077341	*TNFRSF10C*	tumor necrosis factor receptor superfamily, member 10c, decoy without an intracellular domain	intron	0.587
rs2793434	*GPLD1*	glycosylphosphatidylinositol specific phospholipase D1	intron	0.587
rs6599300	*MAEA*	macrophage erythroblast attacher	intron	0.584
rs13340461	*CCND3*	cyclin D3	intron	0.584
rs11784487	*ANK1*	Ankyrin 1	intron	0.584
rs10849446	*SCNN1A*	sodium channel, nonvoltage-gated 1 alpha	intron	0.583
rs12186418	*PDZD2*	PDZ domain containing 2	intron	0.583

**^a^**For nonsynonymous substitutions the aminoacid change is reported.

SNPs are ranked according to τ values. For multiple correlating SNPs in the same gene, the correlation coefficient is only shown for the strongest SNP.

Previous works have reported an enrichment of selection signatures within or in close proximity to human genes [12],[35]. In line with these data we verified that virus-associated SNPs are more frequently located within gene regions compared to a control set of MAF-matched variants (χ^2^ test, *p* = 0.026).

### Functional characterization of genes subjected to virus-driven selective pressure

**Figure 1:**
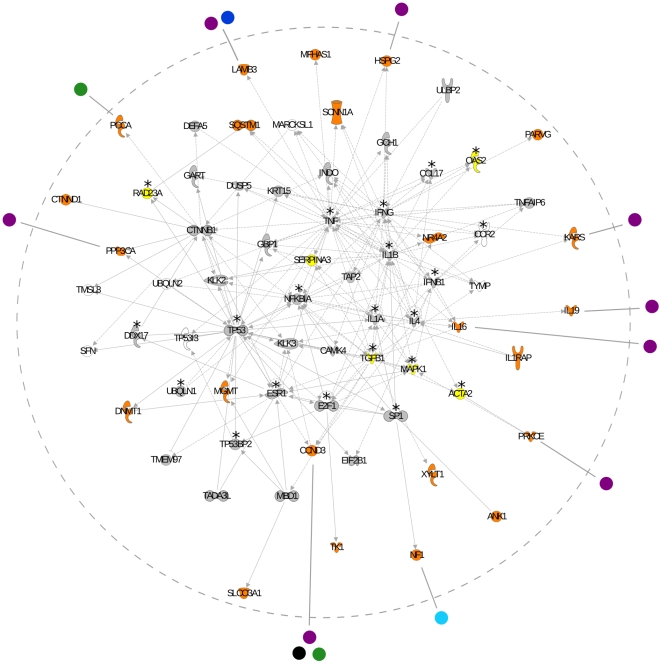
Network analysis of genes associated with virus diversity. Interactions between human proteins are delimited by the hatched grey circle. Genes are represented as nodes; edges indicate known interactions (sold lines depicts direct and hatched lines depict indirect interaction). Human genes are colour-coded as follows: orange, genes with at least one SNP significantly associated with virus diversity; yellow, genes with at least one SNP that did not withstand genome-wide Bonferroni correction but displayed a rank higher than the 99^th^ and a *p* value lower than 10^−5^ (these genes were not included in the input IPA list used to generate networks); grey, genes covered by at least one SNP in the HGDP-CEPH panel; white, genes with no SNPs in the panel. Virus-host interactions are shown for genes subjected to virus-driven selection only; genes interacting with viral products that display no SNP significantly associated with virus diversity are denoted with an asterisk. Viral products are reported outside the hatched circle and colour coded as follows: purple, HIV-1; green, Human herpesvirus; blue, Human rotavirus G3; cyan, Human adenovirus 2; black, Human T-lymphotropic virus 1.

We investigated the role and functional relationship among genes subjected to virus-driven selective pressure using the Ingenuity Pathway Analysis (IPA, Ingenuity Systems) and the PANTHER classification system [36]–[37]. Unsupervised IPA analysis retrieved two networks with significant scores (*p* = 10^−17^ and *p* = 10^−12^) which were merged into a single interaction network (Figure 1). The network contains 23 genes showing a significant correlation with virus diversity and, among these, 10 encode proteins interacting with viral products (Figure 1). Based on the number of observed human-virus interactions, this finding is unlikely to occur by chance (χ^2^ test, *p* = 0.0013) as 2.88 human-virus interactions would be expected for 23 genes. Analysis of the whole network indicated that a 31 of 66 genes encode proteins interacting with viral products (Figure 1): again this number is higher than expected (expected interactions  = 8.27; χ^2^ test, *p* = 2.8×10^−10^). Thus, the interaction network we have identified is enriched in genes subjected to virus-driven selective pressure and in genes coding for proteins interacting with viral products. It is worth mentioning that, in agreement with previous findings [38], many viral-interacting proteins represent hubs in the network. Conversely, most of the genes we found to be subjected to virus-driven selective pressure, irrespective of their ability to interact with viral proteins, tend to display very low connectivity (low-degree nodes). This observation might be consistent with previous indications [39]–[41] that in eukaryotes hub genes are more selectively constrained compared to low-degree nodes, these latter being more likely to evolve in response to environmental pressures.

In addition to proteins directly interacting with viral products, several network genes showing correlation with virus diversity might play central roles during viral infection. *DNMT1* (MIM 126375) and *MGMT* (MIM 156569) are involved in DNA methylation and repair, respectively, two processes that are often dysregulated during viral infection. In particular, altered expression of *DNMT1* is induced by diverse viruses including HIV-1 [42], EBV [43], BKV and adenovirsuses [44]; also, *DNMT1* plays a pivotal role in the expansion of effector CD8+ T cell following viral infection [45]. A relevant role in HIV-1 infection is also played by *HSPG2* (MIM 142461), the gene coding for perlecan, a cell surface heparan sulfate proteoglycan which mediates the internalization of Tat protein [46].

We next investigated the over-representation of PANTHER classification categories among genes subjected to virus-driven selective pressure. Table 4 shows the significantly over-represented PANTHER molecular functions and biological processes with the contributing genes. In line with the results we reported above, genes involved in immune response, as well as genes coding for proteins involved in cell adhesion and extracellular matrix components, resulted to be over-represented; these latter genes might mediate viral-cellular interaction and facilitate viral entry.

**Table 4:** Significantly over-represented PANTHER categories.

PANTHER category	PANTHER description	Number of genes^a^	*p* value^b^
**Biological process**	Signal transduction	61	1.74×10^−9^
	Cell adhesion-mediated signalling	16	9.10×10^−6^
	Cell adhesion	16	7.98×10^−4^
	Cell communication	24	2.79×10^−3^
	Neuronal activities	13	1.43×10^−2^
	Carbohydrate metabolism	13	2.05×10^−2^
	Extracellular matrix protein-mediated signalling	5	2.47×10^−2^
	Immunity and defense	21	3.56×10^−2^
**Molecular function**	Receptor	30	4.27×10^−5^
	Other receptor	11	3.19×10^−4^
	Extracellular matrix linker protein	4	5.27×10^−3^
	Extracellular matrix	10	2.29×10^−2^

**^a^**Number of genes that correlate with virus diversity in each PANTHER category.

**^b^** *p* values are Bonferroni corrected.

## Discussion

The identification of non-neutrally evolving loci with a role in immunity can be regarded as a strategy complementary to classic clinical and epidemiological studies in providing insight into the mechanisms of host defense [47]. Here we propose that susceptibility genes for viral infections can be identified by searching for SNPs that display a strong correlation with the diversity of virus species/genera transmitted in different geographic areas. Similar approaches have previously been applied to study the adaptation to climate for genes involved in metabolism and sodium handling [48]–[50]. These analyses, including the one we describe herein, rely on similar assumptions and imply some caveats. First, we implicitly considered virus diversity, as we measure it nowadays, a good proxy for long-term selective pressure. This clearly represents an oversimplification, as new viral pathogens have recently emerged and the virulence of different viral species or genera might have changed over time. Still, previous studies have indicated that the geographic distribution of virus diversity is strongly influenced by climatic variables such as temperature and precipitation rates [10], suggesting that, despite significant changes in prevalence and virulence, virus diversity might have remained relatively constant across different geographic areas, possibly representing the best possible estimate of long-standing pressure. In line with these considerations, we calculated virus diversity as the number of all viral species (or genera/families) that can cause a disease in humans, irrespective of virulence or pathogenicity (Table S6).

The second issue relevant to the data we present herein is that environmental variables tend to co-vary across geographic regions: the distribution of different pathogens (e.g. parasitic worms and viruses/bacteria/protozoa) is correlated across HGDP-CEPH populations [9] and, as reported above, virus diversity is influenced by climatic factors. Therefore, our genome-wide search was preceded by analyses aimed at verifying whether virus diversity is a reliable and specific estimator of virus-driven selective pressure. In particular, we verified that genes involved in immune response and in the biosynthesis of glycans display significantly more variants associated with virus diversity than randomly selected human genes; this finding supports the idea that pathogens rather than climate or demography has driven the genetic variability at these loci. Notably, we also analysed genes that encode proteins interacting with viral components: since loci involved in immune response and in glycan biosynthesis were removed from this list, the remaining genes are expected to be specific targets of viral-driven selective pressure; consistently, we verified that a significant excess of SNPs correlating with virus diversity map to these loci. Conversely, a SNP excess was not noticed when the diversity of other human pathogens was used for the analysis, suggesting that, despite the correlation among different pathogen species across geographic locations [9], the selective pressure imposed by viruses can be distinguished from that exerted by other organisms.

As a further control for the possible confounding effects of other environmental factors, we verified that the variants we identified at the genome-wide level do not correlate with climate (temperature) and UV radiation. This analysis was motivated by the known association of virus diversity and biodiversity in general, with temperature [10],[51] and by the fact that both climate and UV exposure have long been considered among the strongest selective pressures in humans [52]. Since none of the SNPs we identified correlated with either short wave radiation flux or temperature, we consider that their geographic distribution is likely to have been shaped by virus-driven selective pressure. In this respect it is worth mentioning that UV irradiation has been shown to be immunosuppressive in mice (reviewed in [53]–[54]), but the effect of sun exposure on immune functions in humans is still poorly understood. Yet, herpes viruses (both simplex and zoster) and some papillomavirus types have been shown to be reactivated by UV exposure, suggesting that the link between short wave radiation flux and virus-driven selective pressure might be more complex than simply predicted on the basis of geographic variation.

Our genome wide search for genes subjected to virus-driven selection allowed the identification of a gene interaction network that is enriched in both genes associated with virus diversity and in genes encoding proteins that interact with viral products. Many of the genes included in the identified network are of great interest as they are known to be involved in the activation of mechanisms that have direct or indirect protective effects against viruses. Thus, beside the well known activities of *IL1A* (MIM 147760) and *B* (MIM 147720), *IL4* (MIM 147780), *TGFB1* (MIM 190180), *IL16* (MIM 603035), *IFNG* (MIM 147570) and *TNF* (MIM 191160), *OAS2* (MIM 603350) encodes a protein that activates latent RNases, resulting in the degradation of viral RNA and in the inhibition of viral replication [55]. CCL17 (MIM 601520) induces T lymphocytes chemotaxis, thus potentiating the immune responses, and PPP3CA (MIM 114105), also known as calcineurin, activates NFATc [56], a key factor in the up-regulation of IL2 (MIM 147680) [57], the main cytokine responsible for T lymphocytes growth and differentiation. Finally, *ULBP2* (MIM 605698) encodes an MHC1-related protein that binds to NKG2D (MIM 602893) [58], an activating receptor expressed on CD8 T cells as well as on NK cells, NKT cells and γδ T cells. In the light of the viral pathogenesis of a growing number of neoplasia, it is very interesting that other members of the network play a well described role in the inhibition of tumoral growth. In particular, *E2F1* (MIM 189971) is known to have a pivotal role in the control of cell cycle and in the activation of tumour suppressor proteins and, together with TP53I3, TADA3L, and TP53BP2 mediates p53-dependent and independent apoptosis [59]–[60]. *CCND3* (MIM 123834) is involved in cell cycle progression through the G2 phase, whereas *RAD23A* (MIM 600061) up-regulates the nucleotide excision activity of 3-methyladenine-DNA glycosylase [61], therefore playing a role in DNA damage recognition in base excision repair. Finally, *NR4A2* (MIM 601828) encodes a nuclear orphan receptor expressed in T cells and involved in apoptosis [62]. *NR4A2* is also known to play a central role in eliciting the production of inflammatory cytokines in multiple sclerosis (MS (MIM 126200)) [63]. Notably, variants in *PPP3CA* (Figure 1) have recently been reported to correlate with MS severity as well [64]. We therefore investigated whether other genes carrying SNPs which correlate with virus diversity have been identified in GWAS for MS susceptibility or severity. Three additional genes, *JMJD2C* (MIM 605469), *C20orf133* (also known as *MACROD2*, (MIM 611567)) and *CSMD1* (MIM 608397) have been associated with MS [64] and display SNPs significantly correlated with virus diversity (Table S1). While the function of *C20orf133* is unknown, *JMJD2C* encodes a histone demethylase expressed at very high levels in B cells and cytotoxic lymphocytes (see materials and methods), a pattern consistent with its being subjected to virus-driven selective pressure. Finally, *CSMD1*, in analogy to the aforementioned *SERPING1*, acts as a regulator of the complement system [65]; notably, complement activation plays a central role in both response to viruses and inflammatory reactions, particularly in the central nervous system [66].

Analysis of the 30 stronger associations (Table 3) indicated that several genes are part of the network described above or have been involved in immune response (see InnateDB gene list, Table 2). Conversely, others encode relatively unknown products (e.g. *KIAA1529* (MIM 611258), *LHFPL3* (MIM 609719), *LOC51149*, *RNF217*, *TMEM132B*, *LEPREL1* (MIM 610341), *ANKFN1*, *MYO5C* (MIM 610022), *ANXA4* (MIM 106491) and *SCRN3*). Among these genes, *MYO5C*, *ANXA4* and *SCRN3* are involved in membrane trafficking events along exocytotic and endocytotic pathways, suggesting that they might play a role in either viral cell entry [67] or lytic granule exocytosis; this might be the case for *ANXA4* which is expressed at high levels in NK cells (see materials and methods). Most interestingly, *EYA4* (MIM 603550) (Table 3) has recently been described as a phosphatase involved in triggering innate immune responses against viruses [68]. Finally, both *PDE2A* (MIM 602658) and *SCNN1A* (MIM 600228) might play a role in maintaining lung epithelial barrier homoeostasis during viral infection. Indeed, both genes can be induced by TNF-alpha in lung epithelial cells [69]–[70] and can influence lung fluid reabsorption and, therefore, edema formation. In line with these observations, expression of the amiloride-sensitive epithelial Na+ channel (*SCNN1A* codes for the α subunit) is affected by infection with influenza virus, severe acute respiratory syndrome coronavirus and respiratory syncitial virus.

In humans, resistance to infectious diseases is thought to be under complex, multigenic control with single loci playing a small protective role [47]. This concept also holds for viral infection as demonstrated by the role of genetic variants in modulating the susceptibility to HIV infection or disease progression (reviewed in [71]). Classic GWAS offer a powerful resource to identify susceptibility loci for infectious diseases; yet GWAS typically have limited power to detect variants with a low frequency or a small effect. Indeed, recent GWAS for SNPs determining the host control of HIV-1 [4]–[5] failed to identify most known loci with a role in AIDS progression. The alternative approach we have proposed here is based on the identification of variants subjected to virus-driven selective pressure. Similarly to the GWAS results mentioned above we did not identify well known antiviral-response genes. Still, we noticed that variants in *TRIM5* (MIM 608487) (rs2291845, τ = 0.44, *p* = 1.86×10^−5^, rank = 0.97) and *IFIH1* (MIM 606951) (also known as *MDA5*, rs10439256, τ = 0.51, *p* = 5.4×10^−7^, rank = 0.99) showed significant associations with virus-diversity, although they did not withstood genome-wide analysis. Also, it is worth mentioning that variants with a well established role in resistance to viral infections may be neutrally evolving; this is the case for the Δ32 allele of *CCR5* (MIM 601373) for example, which confers protection against HIV-1 infection and possibly against other pathogens, but displays no selection signature [72]. This is possibly due to how long and how strong the selective pressure has been exerted. Conversely, variants subjected to selective pressure must have (or have had along human history) some selective advantage, indicating that the SNPs we have identified can be regarded as candidate modulators of infection susceptibility or disease progression.

## Materials and Methods

### Environmental variables

Virus absence/presence matrices for the 21 countries where HGDP-CEPH populations are located were derived from the Global Infectious Disease and Epidemiology Network database (Gideon, http://www.gideononline.com), a global infectious disease knowledge tool. Information in Gideon is weekly updated and derives from World Health Organization reports, National Health Ministries, PubMed searches and epidemiology meetings. The Gideon Epidemiology module follows the status of known infectious diseases globally, as well as in individual countries, with specific notes indicating the disease's history, incidence and distribution per country. We manually curated virus absence/presence matrices by extracting information from single Gideon entries. These may refer to either species, genera or families (in case data are not available for different species of a same genus/family). Following previous suggestions [7]–[9], we recorded only viruses that are transmitted in the 21 countries, meaning that cases of transmission due to tourism and immigration were not taken into account; also, species that have recently been eradicated as a result, for example, of vaccination campaigns, were recorded as present in the matrix. A total of 81 virus species/genera/families were retrieved (Table S6). The same approach was applied to calculate the diversity of other pathogens, namely bacteria, protozoa and helminths [9]. The annual minimum and maximum temperature were retrieved from the NCEP/NCAR database (http://www.ngdc.noaa.gov/ecosys/cdroms/ged_iia/datasets/a04/, Legates and Willmott Average, re-gridded dataset) using the geographic coordinates reported by HGDP-CEPH website for each population (http://www.cephb.fr/en/hgdp/table.php). Similarly, net short wave radiation flux data were obtained from NCEP/NCAR (http://www.esrl.noaa.gov/psd/data/gridded/data.ncep.reanalysis.surfaceflux.html, Reanalysis 1: Surface Flux); these data were read using Grid Analysis and Display System (GrADS, http://www.iges.org/grads/). Daily values for four years (1948–1951) were averaged to obtain an annual mean.

Since virus diversity, due to data organization in Gideon, can only be calculated per country (rather than per population), the same procedure was applied to climatic variables. Therefore the values of annual temperature and radiation flux were averaged for populations located in the same country. This assures that a similar number of ties is maintained in all correlation analyses.

### Data retrieval and statistical analysis

Data concerning the HGDP-CEPH panel derive from a previous work [6]. Atypical or duplicated samples and pairs of close relatives were removed [73].

A SNP was ascribed to a specific gene if it was located within the transcribed region or no farther than 500 bp upstream the transcription start site. MAF for any single SNP was calculated as the average over all populations. The list of immune response genes was derived from the InnateDB website (http://www.innatedb.com/) and it contains a non-redundant list of 5,070 immune genes derived from ImmPort, IRIS, Septic Shock Group, MAPK/NFKB Network and Immunome Database; it only includes genes derived from curated immune gene lists.

Genes involved in glycan biosynthesis were obtained by merging genes from two KEGG pathways (“Glycan structures - biosynthesis 1” and “Glycan structures - biosynthesis 2”). Additional genes were identified by searching Gene Ontology categories for genes that act as glycosyltransferases (GO:0016757) and are located in either the Golgi or the endoplasmic reticulum (GO:0005783, GO:0005793 and GO:0005794). The list of human genes coding for proteins interacting with viral products was derived from three sources: a previously published study [38], the VirHostNet website [74] (http://pbildb1.univ-lyon1.fr/virhostnet/) and the HIV-1 Human Protein Interaction Database [75] (http://www.ncbi.nlm.nih.gov/RefSeq/HIVInteractions/).

Expression data were obtained from SymAtlas (http://symatlas.gnf.org/). The location of genomic elements that are highly conserved among vertebrates was derived from UCSC annotation tables (http://genome.ucsc.edu/; “PhastCons Conserved Elements, 44-way Vertebrate Multiz Alignment” track).

All correlations were calculated by Kendall's rank correlation coefficient (τ), a non-parametric statistic used to measure the degree of correspondence between two rankings. The reason for using this test is that even in the presence of ties, the sampling distribution of τ satisfactorily converges to a normal distribution for values of *n* larger than 10 [76].

In order to estimate the probability of obtaining *n* SNPs located within *m* genes and significantly associated with virus diversity, we applied a re-sampling approach: samples of *m* genes were randomly extracted from a list of all genes covered by at least one SNP in the HGDP-CEPH panel (number of genes  = 15,280) and for each sample the number of SNPs significantly associated with virus diversity was counted. The empirical probability of obtaining *n* SNPs was then calculated from the distribution of counts deriving from 10,000 random samples. A SNP was ascribed to a gene if it was located within the transcribed region or in the 500 upstream nucleotides.

Analysis of PANTHER over-represented functional categories and pathways was performed using the “Compare Classifications of Lists” tool available at the PANTHER classification system website [77] (http://www.pantherdb.org/). Briefly, gene lists are compared to the reference list using the binomial test for each molecular function, biological process, or pathway term in PANTHER.

All calculation were performed in the R environment [78] (http://www.r-project.org/).

### Network construction

Biological network analysis was performed with Ingenuity Pathways Analysis (IPA) software using an unsupervised analysis (www.ingenuity.com). IPA builds networks by querying the Ingenuity Pathways Knowledge Base for interactions between the identified genes and all other gene objects stored in the knowledge base; it then generates networks with a maximum network size of 35 genes/proteins. We used all genes showing at least one significantly associated SNP as the input set; in this case a SNP was ascribed to a gene if it was located within the transcribed region or in the 25 kb upstream. All network edges are supported by at least one published reference or from canonical information stored in the Ingenuity Pathways Knowledge Base. To determine the probability of the analysed genes to be found together in a network from Ingenuity Pathways Knowledge Base due to random chance alone, IPA applies a Fisher's exact test. The network score represents the -log (*p* value).

## Supporting Information

Table S1 SNPs in InnateDB genes that significantly correlate with virus diversity.
(0.05 MB PDF)

Table S2 SNPs in genes involved in glycan biosynthesis that significantly correlate with virus diversity.
(0.03 MB DOC)

Table S3 SNPs in genes coding for proteins interacting with viral products that significantly correlate with virus diversity.
(0.05 MB PDF)

Table S4 SNPs significantly associated with virus diversity. The table reports all SNPs that withstood Bonferroni correction at the genome-wide level (with α = 0.05) and displayed a Tau percentile rank higher than the 99^th^ among MAF-matched SNPs, as described in the main text and in material and methods. SNPs are ranked according to the value of Tau. If the SNP is located within a genic region (or in the 500 upstream nucleotides) the gene symbol is reported. Also, the gene closest to the SNP and its distance (in bp) are indicated. The aminoacid substitution is reported for nonsynonymous variants; SNPs annotated as “phastCons element” are located within non-coding genomic regions that display high sequence conservation among mammals (as described in the text).
(0.21 MB DOC)

Table S5 Correlations between SNPs associated with virus diversity and other climatic variables. The table shows correlation coefficients between each SNP associated with virus diversity and the following climatic variables: average annual maximum temperature (Tmax), average annual minimum temperature (Tmin), short wave radiation flux (Irradiation SW). After Bonferroni correction all *p* values were >0.05.
(0.42 MB DOC)

Table S6 List of viruses identified in at least one country (n = 81)
(0.01 MB DOC)

## References

[1] Morens DM, Folkers GK, Fauci AS (2004). The challenge of emerging and re-emerging infectious diseases.. Nature.

[2] Lander ES, Linton LM, Birren B, Nusbaum C, Zody MC (2001). Initial sequencing and analysis of the human genome.. Nature.

[3] Beutler B, Eidenschenk C, Crozat K, Imler JL, Takeuchi O (2007). Genetic analysis of resistance to viral infection.. Nat Rev Immunol.

[4] Limou S, Le Clerc S, Coulonges C, Carpentier W, Dina C (2009). Genomewide association study of an AIDS-nonprogression cohort emphasizes the role played by HLA genes (ANRS genomewide association study 02).. J Infect Dis.

[5] Fellay J, Shianna KV, Ge D, Colombo S, Ledergerber B (2007). A whole-genome association study of major determinants for host control of HIV-1.. Science.

[6] Li JZ, Absher DM, Tang H, Southwick AM, Casto AM (2008). Worldwide human relationships inferred from genome-wide patterns of variation.. Science.

[7] Prugnolle F, Manica A, Charpentier M, Guegan JF, Guernier V (2005). Pathogen-driven selection and worldwide HLA class I diversity.. Curr Biol.

[8] Fumagalli M, Cagliani R, Pozzoli U, Riva S, Comi GP (2009). Widespread balancing selection and pathogen-driven selection at blood group antigen genes.. Genome Res.

[9] Fumagalli M, Pozzoli U, Cagliani R, Comi GP, Riva S (2009). Parasites represent a major selective force for interleukin genes and shape the genetic predisposition to autoimmune conditions.. J Exp Med.

[10] Guernier V, Hochberg ME, Guegan JF (2004). Ecology drives the worldwide distribution of human diseases.. PLoS Biol.

[11] Handley LJ, Manica A, Goudet J, Balloux F (2007). Going the distance: Human population genetics in a clinal world.. Trends Genet.

[12] Coop G, Pickrell JK, Novembre J, Kudaravalli S, Li J (2009). The role of geography in human adaptation.. PLoS Genet.

[13] Yang B, Chen K, Zhang C, Huang S, Zhang H (2007). Virion-associated uracil DNA glycosylase-2 and apurinic/apyrimidinic endonuclease are involved in the degradation of APOBEC3G-edited nascent HIV-1 DNA.. J Biol Chem.

[14] Izmailova E, Bertley FM, Huang Q, Makori N, Miller CJ (2003). HIV-1 tat reprograms immature dendritic cells to express chemoattractants for activated T cells and macrophages.. Nat Med.

[15] Gerencer M, Burek V (2004). Identification of HIV-1 protease cleavage site in human C1-inhibitor.. Virus Res.

[16] Drouet C, Bouillet L, Csopaki F, Colomb MG (1999). Hepatitis C virus NS3 serine protease interacts with the serpin C1 inhibitor.. FEBS Lett.

[17] Dronamraju K (1990). Selected genetic papers of J.B.S. haldane..

[18] Imberty A, Varrot A (2008). Microbial recognition of human cell surface glycoconjugates.. Curr Opin Struct Biol.

[19] Erbacher A, Gieseke F, Handgretinger R, Muller I (2009). Dendritic cells: Functional aspects of glycosylation and lectins.. Hum Immunol.

[20] Van Dyken SJ, Green RS, Marth JD (2007). Structural and mechanistic features of protein O glycosylation linked to CD8+ T-cell apoptosis.. Mol Cell Biol.

[21] Srinivasan A, Viswanathan K, Raman R, Chandrasekaran A, Raguram S (2008). Quantitative biochemical rationale for differences in transmissibility of 1918 pandemic influenza A viruses.. Proc Natl Acad Sci U S A.

[22] Chandrasekaran A, Srinivasan A, Raman R, Viswanathan K, Raguram S (2008). Glycan topology determines human adaptation of avian H5N1 virus hemagglutinin.. Nat Biotechnol.

[23] Neu U, Stehle T, Atwood WJ (2009). The polyomaviridae: Contributions of virus structure to our understanding of virus receptors and infectious entry.. Virology.

[24] Isa P, Arias CF, Lopez S (2006). Role of sialic acids in rotavirus infection.. Glycoconj J.

[25] Zeng J, Joo HM, Rajini B, Wrammert JP, Sangster MY (2009). The generation of influenza-specific humoral responses is impaired in ST6Gal I-deficient mice.. J Immunol.

[26] Avril T, North SJ, Haslam SM, Willison HJ, Crocker PR (2006). Probing the cis interactions of the inhibitory receptor siglec-7 with alpha2,8-disialylated ligands on natural killer cells and other leukocytes using glycan-specific antibodies and by analysis of alpha2,8-sialyltransferase gene expression.. J Leukoc Biol.

[27] Shukla D, Spear PG (2001). Herpesviruses and heparan sulfate: An intimate relationship in aid of viral entry.. J Clin Invest.

[28] Lambert S, Bouttier M, Vassy R, Seigneuret M, Petrow-Sadowski C (2009). HTLV-1 uses HSPG and neuropilin-1 for entry by molecular mimicry of VEGF165.. Blood.

[29] Johnson KM, Kines RC, Roberts JN, Lowy DR, Schiller JT (2009). Role of heparan sulfate in attachment to and infection of the murine female genital tract by human papillomavirus.. J Virol.

[30] Mardberg K, Trybala E, Tufaro F, Bergstrom T (2002). Herpes simplex virus type 1 glycoprotein C is necessary for efficient infection of chondroitin sulfate-expressing gro2C cells.. J Gen Virol.

[31] Argyris EG, Acheampong E, Nunnari G, Mukhtar M, Williams KJ (2003). Human immunodeficiency virus type 1 enters primary human brain microvascular endothelial cells by a mechanism involving cell surface proteoglycans independent of lipid rafts.. J Virol.

[32] Rojek JM, Spiropoulou CF, Campbell KP, Kunz S (2007). Old world and clade C new world arenaviruses mimic the molecular mechanism of receptor recognition used by alpha-dystroglycan's host-derived ligands.. J Virol.

[33] Kunz S, Rojek JM, Kanagawa M, Spiropoulou CF, Barresi R (2005). Posttranslational modification of alpha-dystroglycan, the cellular receptor for arenaviruses, by the glycosyltransferase LARGE is critical for virus binding.. J Virol.

[34] Sabeti PC, Varilly P, Fry B, Lohmueller J, Hostetter E (2007). Genome-wide detection and characterization of positive selection in human populations.. Nature.

[35] Barreiro LB, Laval G, Quach H, Patin E, Quintana-Murci L (2008). Natural selection has driven population differentiation in modern humans.. Nat Genet.

[36] Thomas PD, Campbell MJ, Kejariwal A, Mi H, Karlak B (2003). PANTHER: A library of protein families and subfamilies indexed by function.. Genome Res.

[37] Thomas PD, Kejariwal A, Guo N, Mi H, Campbell MJ (2006). Applications for protein sequence-function evolution data: MRNA/protein expression analysis and coding SNP scoring tools.. Nucleic Acids Res.

[38] Dyer MD, Murali TM, Sobral BW (2008). The landscape of human proteins interacting with viruses and other pathogens.. PLoS Pathog.

[39] Albert R (2005). Scale-free networks in cell biology.. J Cell Sci.

[40] Fraser HB, Hirsh AE, Steinmetz LM, Scharfe C, Feldman MW (2002). Evolutionary rate in the protein interaction network.. Science.

[41] Pagel M, Meade A, Scott D (2007). Assembly rules for protein networks derived from phylogenetic-statistical analysis of whole genomes.. BMC Evol Biol.

[42] Youngblood B, Reich NO (2008). The early expressed HIV-1 genes regulate DNMT1 expression.. Epigenetics.

[43] Hino R, Uozaki H, Murakami N, Ushiku T, Shinozaki A (2009). Activation of DNA methyltransferase 1 by EBV latent membrane protein 2A leads to promoter hypermethylation of PTEN gene in gastric carcinoma.. Cancer Res.

[44] McCabe MT, Low JA, Imperiale MJ, Day ML (2006). Human polyomavirus BKV transcriptionally activates DNA methyltransferase 1 through the pRb/E2F pathway.. Oncogene.

[45] Chappell C, Beard C, Altman J, Jaenisch R, Jacob J (2006). DNA methylation by DNA methyltransferase 1 is critical for effector CD8 T cell expansion.. J Immunol.

[46] Argyris EG, Kulkosky J, Meyer ME, Xu Y, Mukhtar M (2004). The perlecan heparan sulfate proteoglycan mediates cellular uptake of HIV-1 tat through a pathway responsible for biological activity.. Virology.

[47] Quintana-Murci L, Alcais A, Abel L, Casanova JL (2007). Immunology in natura: Clinical, epidemiological and evolutionary genetics of infectious diseases.. Nat Immunol.

[48] Hancock AM, Witonsky DB, Gordon AS, Eshel G, Pritchard JK (2008). Adaptations to climate in candidate genes for common metabolic disorders.. PLoS Genet.

[49] Thompson EE, Kuttab-Boulos H, Witonsky D, Yang L, Roe BA (2004). CYP3A variation and the evolution of salt-sensitivity variants.. Am J Hum Genet.

[50] Young JH, Chang YP, Kim JD, Chretien JP, Klag MJ (2005). Differential susceptibility to hypertension is due to selection during the out-of-africa expansion.. PLoS Genet.

[51] Allen AP, Brown JH, Gillooly JF (2002). Global biodiversity, biochemical kinetics, and the energetic-equivalence rule.. Science.

[52] Novembre J, Di Rienzo A (2009). Spatial patterns of variation due to natural selection in humans.. Nat Rev Genet.

[53] Sleijffers A, Garssen J, Van Loveren H (2002). Ultraviolet radiation, resistance to infectious diseases, and vaccination responses.. Methods.

[54] Norval M (2006). The effect of ultraviolet radiation on human viral infections.. Photochem Photobiol.

[55] Justesen J, Hartmann R, Kjeldgaard NO (2000). Gene structure and function of the 2′-5′-oligoadenylate synthetase family.. Cell Mol Life Sci.

[56] Crabtree GR, Olson EN (2002). NFAT signaling: Choreographing the social lives of cells.. Cell.

[57] Shaw JP, Utz PJ, Durand DB, Toole JJ, Emmel EA (1988). Identification of a putative regulator of early T cell activation genes.. Science.

[58] Sutherland CL, Chalupny NJ, Schooley K, VandenBos T, Kubin M (2002). UL16-binding proteins, novel MHC class I-related proteins, bind to NKG2D and activate multiple signaling pathways in primary NK cells.. J Immunol.

[59] Sherr CJ (1998). Tumor surveillance via the ARF-p53 pathway.. Genes Dev.

[60] Irwin M, Marin MC, Phillips AC, Seelan RS, Smith DI (2000). Role for the p53 homologue p73 in E2F-1-induced apoptosis.. Nature.

[61] Miao F, Bouziane M, Dammann R, Masutani C, Hanaoka F (2000). 3-methyladenine-DNA glycosylase (MPG protein) interacts with human RAD23 proteins.. J Biol Chem.

[62] Cheng LE, Chan FK, Cado D, Winoto A (1997). Functional redundancy of the Nur77 and nor-1 orphan steroid receptors in T-cell apoptosis.. EMBO J.

[63] Doi Y, Oki S, Ozawa T, Hohjoh H, Miyake S (2008). Orphan nuclear receptor NR4A2 expressed in T cells from multiple sclerosis mediates production of inflammatory cytokines.. Proc Natl Acad Sci U S A.

[64] Baranzini SE, Wang J, Gibson RA, Galwey N, Naegelin Y (2009). Genome-wide association analysis of susceptibility and clinical phenotype in multiple sclerosis.. Hum Mol Genet.

[65] Kraus DM, Elliott GS, Chute H, Horan T, Pfenninger KH (2006). CSMD1 is a novel multiple domain complement-regulatory protein highly expressed in the central nervous system and epithelial tissues.. J Immunol.

[66] Speth C, Dierich MP, Gasque P (2002). Neuroinvasion by pathogens: A key role of the complement system.. Mol Immunol.

[67] Mercer J, Helenius A (2009). Virus entry by macropinocytosis.. Nat Cell Biol.

[68] Okabe Y, Sano T, Nagata S (2009). Regulation of the innate immune response by threonine-phosphatase of eyes absent.. Nature.

[69] Dagenais A, Frechette R, Clermont ME, Masse C, Prive A (2006). Dexamethasone inhibits the action of TNF on ENaC expression and activity.. Am J Physiol Lung Cell Mol Physiol.

[70] Seybold J, Thomas D, Witzenrath M, Boral S, Hocke AC (2005). Tumor necrosis factor-alpha-dependent expression of phosphodiesterase 2: Role in endothelial hyperpermeability.. Blood.

[71] Piacentini L, Biasin M, Fenizia C, Clerici M (2009). Genetic correlates of protection against HIV infection: The ally within.. J Intern Med.

[72] Sabeti PC, Walsh E, Schaffner SF, Varilly P, Fry B (2005). The case for selection at CCR5-Delta32.. PLoS Biol.

[73] Rosenberg NA (2006). Standardized subsets of the HGDP-CEPH human genome diversity cell line panel, accounting for atypical and duplicated samples and pairs of close relatives.. Ann Hum Genet.

[74] Navratil V, de Chassey B, Meyniel L, Delmotte S, Gautier C (2009). VirHostNet: A knowledge base for the management and the analysis of proteome-wide virus-host interaction networks.. Nucleic Acids Res.

[75] Fu W, Sanders-Beer BE, Katz KS, Maglott DR, Pruitt KD (2009). Human immunodeficiency virus type 1, human protein interaction database at NCBI.. Nucleic Acids Res.

[76] Salkind NJ (2007). Encyclopedia of measurement and statistics..

[77] Cho RJ, Campbell MJ (2000). Transcription, genomes, function.. Trends Genet.

[78] R Development Core Team (2008). R: A language and environment for statistical computing. Vienna, Austria..

