# Supplementary material for: Genome-Wide Identification of Susceptibility Alleles for Viral Infections through a Population Genetics Approach
Source: PLoS Genet. 2010 Feb 19;6(2):e1000849. doi: 10.1371/journal.pgen.1000849 (PMC2824813; doi:10.1371/journal.pgen.1000849)
Supplement: Table S1 — SNPs in InnateDB genes that significantly correlate with virus diversity. (0.05 MB PDF) [file pgen.1000849.s001.pdf]

Table S1. SNPs in InnateDB genes that significantly correlate with virus diversity.

SNP	Gene	Annotation[a]	τ	*p* value
rs993715	*CNTNAP2*	intron	0.6085	0.00015260
rs2189883	*CNTNAP2*	intron	0.6081	0.00014171
rs3785415	*CDH15*	intron	0.6032	0.00016120
rs4575989	*C1QTNF7*	intron	0.5965	0.00022806
rs7637370	*CLDN18*	intron	0.5960	0.00023918
rs4629443	*C1QTNF7*	intron	0.5955	0.00023966
rs7927476	*NELL1*	intron	0.5934	0.00028950
rs1065154	*SQSTM1*	3' UTR	0.5893	0.00040041
rs12145973	*IL19*	intron	0.5890	0.00310987
rs4953260	*PRKCE*	intron	0.5872	0.00043864
rs4077341	*TNFRSF10C*	intron	0.5869	0.00044225
rs2793434	*GPLD1*	intron	0.5866	0.00040744
rs6599300	*MAEA*	intron	0.5843	0.00051153
rs13340461	*CCND3*	intron	0.5840	0.00062923
rs10849446	*SCNN1A*	intron	0.5831	0.00055910
rs12186418	*PDZD2*	intron	0.5831	0.00049477
rs4698103	*C1QTNF7*	intron	0.5825	0.00051729
rs17282579	*CLDN18*	intron	0.5806	0.00072735
rs2600306	*CNTN4*	intron	0.5800	0.00065050
rs700550	*LRP2*	intron	0.5777	0.00074769
rs971403	*LAMA4*	intron	0.5776	0.00087172
rs2659501	*PPP3CA*	intron	0.5771	0.00075242
rs2016977	*ST8SIA1*	intron	0.5738	0.00281559
rs3782525	*ST8SIA1*	intron	0.5738	0.00281559
rs4130023	*CCND3*	intron, phastCons element	0.5725	0.00120410
rs1346690	*NELL1*	intron	0.5724	0.00099399
rs2527049	*CNTNAP2*	intron	0.5719	0.00138919
rs5917027	*CLDN2*	intron	0.5718	0.00194824
rs1874108	*FREM1*	intron, phastCons element	0.5711	0.00117500
rs1055636	*SLFN5*	3' UTR	0.5705	0.00177587
rs2714174	*LRP1B*	intron	0.5702	0.00118573
rs1402470	*LRP1B*	intron	0.5685	0.00140795
rs1922889	*CNTNAP2*	intron	0.5682	0.00156774
rs11242715	*GMDS*	intron	0.5661	0.00167284
rs7712010	*PDZD2*	intron	0.5659	0.00150229

rs17656058	*CLEC4F*	intron, phastCons element	0.5655	0.00279468
rs708228	*CTNND1*	3' UTR, phastCons element	0.5647	0.00210315
rs13182372	*PDZD2*	intron	0.5646	0.00171276
rs4698374	*C1QTNF7*	intron	0.5642	0.00151817
rs2683824	*LRP1B*	intron	0.5629	0.00179731
rs2501254	*HSPG2*	intron	0.5595	0.00191694
rs12568035	*LAMB3*	intron	0.5575	0.00322326
rs2275254	*CHIA*	F354S	0.5571	0.00220021
rs3856982	*MAEA*	intron	0.5565	0.00249450
rs2850350	*PPP3CA*	intron	0.5564	0.00220599
rs1848116	*PPP3CA*	intron	0.5563	0.00239862
rs7736502	*PDZD2*	intron	0.5559	0.00260144
rs7107376	*FCHSD2*	intron	0.5558	0.00339272
rs2707575	*CNTNAP2*	intron	0.5550	0.00312479
rs1061631	*TNFRSF1B*	3' UTR	0.5547	0.00465174
rs2850976	*PPP3CA*	intron	0.5544	0.00242220
rs1138803	*GMDS*	intron	0.5540	0.00263712
rs953035	*PSMB2*	intron	0.5532	0.00286997
rs980618	*IL16*	intron	0.5524	0.00300278
rs2541886	*UNG*	intron	0.5519	0.00339001
rs12879377	*GALNTL1*	M201V	0.5512	0.00488440
rs10857274	*DCHS2*	intron	0.5506	0.00328931
rs4386	*TOM1*	intron	0.5506	0.00328721
rs7695691	*MAEA*	intron	0.5496	0.00357637
rs565280	*SQSTM1*	intron	0.5490	0.00374946
rs2275603	*FCRLA*	S203G	0.5484	0.00388369
rs372454	*MMD2*	3' UTR	0.5477	0.00392815
rs11771941	*CNTNAP2*	intron	0.5477	0.00487690
rs7121400	*NELL1*	intron	0.5473	0.00410900
rs4782496	*CBFA2T3*	intron	0.5462	0.00483234
rs10502966	*DCC*	intron	0.5453	0.00854755
rs11160322	*BDKRB2*	intron	0.5451	0.00487070
rs6706275	*HDAC4*	intron	0.5435	0.00718766
rs9985234	*LPP*	intron	0.5423	0.00981179
rs2037407	*LIN7A*	intron	0.5423	0.00576535
rs6942912	*CNTNAP2*	intron	0.5421	0.00596000
rs2708285	*CNTNAP2*	intron	0.5420	0.00596000

rs2205084	*DSCAM*	intron	0.5412	0.00605740
rs133399	*TOM1*	intron	0.5410	0.00605864
rs10178342	*LRP1B*	intron	0.5410	0.00539363
rs1351026	*PPFIBP1*	intron	0.5410	0.00539697
rs13329773	*CBFA2T3*	intron	0.5407	0.00677315
rs11049057	*PPFIBP1*	intron	0.5406	0.00607606
rs4411113	*DDR2*	intron	0.5404	0.00608105
rs2583401	*PPP3CA*	intron	0.5402	0.00542488
rs822577	*ARHGEF11*	intron	0.5396	0.00589956
rs10911812	*HMCN1*	intron	0.5396	0.00589231
rs3824988	*SERPING1*	intron	0.5396	0.00894839
rs7954545	*PKP2*	intron	0.5390	0.00638318
rs3217881	*CCND2*	intron	0.5386	0.00689978
rs139149	*PARVG*	intron	0.5382	0.00642763
rs2796813	*TGFB2*	intron	0.5380	0.00722204
rs448849	*CLDN14*	intron	0.5376	0.00698720
rs531071	*NEGR1*	intron, phastCons element	0.5374	0.00751769
rs4651292	*HMCN1*	intron	0.5365	0.00758328
rs183156	*PDZD2*	intron	0.5361	0.00732365
rs419941	*CLDN14*	intron	0.5361	0.00732365
rs870144	*ADARB1*	intron	0.5361	0.00732365
rs8106303	*FXYD5*	intron	0.5349	0.00920391
rs2526121	*SEMA5A*	intron	0.5345	0.00769579
rs878949	*HSPG2*	intron	0.5343	0.00768806
rs10842938	*PPFIBP1*	intron	0.5341	0.00770357
rs3128575	*COL5A1*	3' UTR, phastCons element	0.5335	0.00862800
rs8023564	*AQP9*	intron	0.5333	0.00972604
rs11049046	*PPFIBP1*	intron	0.5325	0.00840894
rs2289239	*POLR1A*	intron	0.5325	0.00975703
rs3773768	*CLDN18*	intron	0.5325	0.00944358
rs1203148	*KIAA0319L*	intron	0.5302	0.00957781
rs12920161	*CDH13*	intron	0.5298	0.00998214

[a]The aminoacid substitution is reported for nonsynonymous SNPs; SNPs annotated as "phastCons element" are located within non-coding genomic regions that display high sequence conservation among mammals (as described in the text).
