# Supplementary material for: Genome-Wide Identification of Susceptibility Alleles for Viral Infections through a Population Genetics Approach
Source: PLoS Genet. 2010 Feb 19;6(2):e1000849. doi: 10.1371/journal.pgen.1000849 (PMC2824813; doi:10.1371/journal.pgen.1000849)
Supplement: Table S2 — SNPs in genes involved in glycan biosynthesis that significantly correlate with virus diversity. (0.03 MB DOC) [file pgen.1000849.s002.doc]

**Table S2. SNPs in genes involved in glycan biosynthesis that significantly correlate with virus diversity.**

| **SNP** | **Gene** | **Annotation^a^** | **τ** | ***p* value** |
| --- | --- | --- | --- | --- |
| rs9989425 | *XYLT1* | intron | 0.5780 | 0.00007856 |
| rs2016977 | *ST8SIA1* | intron | 0.5738 | 0.0002516 |
| rs3782525 | *ST8SIA1* | intron | 0.5738 | 0.0002516 |
| rs7935223 | *GALNTL4* | intron, phastCons element | 0.5738 | 0.0001710 |
| rs2550904 | *CHST6* | intron | 0.5683 | 0.0001123 |
| rs12818044 | *B4GALNT3* | intron | 0.5641 | 0.0008002 |
| rs12879377 | *GALNTL1* | M201V | 0.5512 | 0.0004365 |
| rs1813202 | *UGCG* | intron | 0.5425 | 0.0005737 |
| rs613075 | *MGAT5B* | intron | 0.5382 | 0.0005962 |
| rs10064618 | *GALNT10* | intron | 0.5369 | 0.0006504 |
| rs17271883 | *FUT6* | intron | 0.5363 | 0.0006746 |
| rs778805 | *FUT6* | P124S | 0.5327 | 0.0008378 |
| rs12820379 | *ST8SIA1* | intron | 0.5321 | 0.002147 |
| rs2103187 | *WBSCR17* | intron | 0.5299 | 0.006313 |
| rs13400566 | *GALNT13* | intron | 0.5280 | 0.002649 |
| rs656024 | *MGAT5B* | intron | 0.5280 | 0.001012 |
| rs1982311 | *GALNT13* | intron | 0.5268 | 0.002141 |
| rs12987759 | *GALNT14* | intron | 0.5253 | 0.001156 |
| rs12426898 | *B4GALNT3* | intron | 0.5249 | 0.001248 |
| rs240354 | *LARGE* | intron | 0.5227 | 0.001415 |
| rs2331957 | *GALNTL1* | intron | 0.5222 | 0.007020 |
| rs12570539 | *ST8SIA6* | intron | 0.5182 | 0.002825 |
| rs10208448 | *GALNT13* | intron | 0.5167 | 0.003675 |
| rs9910060 | *MGAT5B* | intron | 0.5152 | 0.001931 |
| rs1352239 | *GALNTL4* | intron | 0.5118 | 0.002627 |
| rs10245251 | *GALNTL5* | intron | 0.5078 | 0.002929 |
| rs7299315 | *GALNT6* | intron | 0.5070 | 0.002944 |
| rs1519213 | *GALNT13* | intron | 0.5045 | 0.003570 |
| rs4747306 | *ST8SIA6* | intron | 0.5028 | 0.004039 |
| rs533097 | *ST6GALNAC3* | intron | 0.5028 | 0.005871 |
| rs12817074 | *ST8SIA1* | intron | 0.5022 | 0.004062 |
| rs3791101 | *ST3GAL3* | intron | 0.5022 | 0.008176 |
| rs12197040 | *UST* | intron | 0.5015 | 0.004532 |
| rs1147465 | *GALNTL1* | intron | 0.5012 | 0.006729 |
| rs13274016 | *TUSC3* | intron | 0.5011 | 0.005387 |
| rs7735907 | *GALNT10* | intron | 0.5011 | 0.004093 |
| rs7929019 | *GALNTL4* | intron | 0.4985 | 0.004952 |
| rs1873588 | *MGAT5B* | intron | 0.4985 | 0.004634 |
| rs3758105 | *ST3GAL1* | intron | 0.4969 | 0.004844 |
| rs6502269 | *HS3ST3A1* | intron | 0.4960 | 0.008540 |
| rs417415 | *ST6GALNAC3* | intron | 0.4953 | 0.005657 |
| rs9860157 | *ST6GAL1* | intron | 0.4941 | 0.006502 |
| rs10212404 | *ST6GAL1* | intron | 0.4938 | 0.006514 |
| rs4910293 | *GALNTL4* | intron | 0.4928 | 0.006587 |
| rs4561106 | *ST8SIA6* | intron | 0.4908 | 0.007156 |
| rs7950646 | *GALNTL4* | intron, phastCons element | 0.4904 | 0.007199 |
| rs3812961 | *XYLT1* | intron | 0.4903 | 0.007718 |
| rs4530793 | *CHSY3* | intron | 0.4894 | 0.009130 |
| rs474589 | *MGAT5B* | intron | 0.4881 | 0.007845 |
| rs240346 | *LARGE* | intron | 0.4864 | 0.009977 |

**^a^**The aminoacid substitution is reported for nonsynonymous SNPs; SNPs annotated as "phastCons element" are located within non-coding genomic regions that display high sequence conservation among mammals (as described in the text).
