# Supplementary material for: Genome-Wide Identification of Susceptibility Alleles for Viral Infections through a Population Genetics Approach
Source: PLoS Genet. 2010 Feb 19;6(2):e1000849. doi: 10.1371/journal.pgen.1000849 (PMC2824813; doi:10.1371/journal.pgen.1000849)
Supplement: Table S3 — SNPs in genes coding for proteins interacting with viral products that significantly correlate with virus diversity. (0.05 MB PDF) [file pgen.1000849.s003.pdf]

Table S3. SNPs in genes coding for proteins interacting with viral products that significantly correlate with virus diversity.

SNP	Gene	Annotation[a]	τ	*p* value
rs1135029	*PDE2A*	A867A	0.6146	0.00002353
rs11235559	*PDE2A*	intron	0.6083	0.00002950
rs189332	*PDE2A*	intron	0.5984	0.00005568
rs1890139	*PCCA*	intron	0.5883	0.00010822
rs10898866	*CENTD2*	intron	0.5753	0.00023944
rs1986656	*TMEM110*	intron	0.5747	0.00096853
rs11150843	*GAA*	R223H	0.5717	0.00027879
rs10793036	*PDE2A*	intron	0.5619	0.00048208
rs10510499	*PCAF*	3' UTR	0.5587	0.00928330
rs2559658	*POLR3A*	intron	0.5583	0.00158061
rs677344	*VPS16*	intron	0.5550	0.00077076
rs2192973	*GRIN2B*	intron	0.5542	0.00079250
rs3784064	*SIPA1L1*	intron	0.5535	0.00135324
rs2525570	*NF1*	intron	0.5506	0.00077971
rs6138953	*PTPRA*	intron	0.5496	0.00377280
rs765688	*GRIN2B*	intron, phastCons element	0.5483	0.00428444
rs1204997	*SIPA1L1*	intron	0.5470	0.00192983
rs1204995	*SIPA1L1*	intron	0.5470	0.00192983
rs133290	*SREBF2*	intron	0.5462	0.00118971
rs2781064	*ENO1*	intron	0.5459	0.00114544
rs341087	*PDE2A*	intron	0.5457	0.00106212
rs2193151	*GRIN2B*	intron	0.5447	0.00511533
rs3784929	*KARS*	intron	0.5447	0.00111157
rs6491544	*PCCA*	intron	0.5445	0.00120365
rs2290559	*SPTBN5*	R1310H	0.5427	0.00222867
rs7318781	*PCCA*	intron	0.5415	0.00148739
rs2783224	*PCCA*	intron	0.5402	0.00155719
rs1339249	*PCSK5*	intron	0.5400	0.00145279
rs1573994	*ITPR2*	intron, phastCons element	0.5390	0.00163913
rs378759	*GTF2E1*	intron	0.5386	0.00152222
rs613075	*MGAT5B*	intron	0.5382	0.00164549
rs9518023	*PCCA*	intron	0.5382	0.00158834
rs2216128	*GRIN2B*	intron	0.5380	0.00171647
rs10493066	*SFPQ*	intron	0.5379	0.00152690

rs1981929	*MSH2*	intron	0.5363	0.00200132
rs9383562	*AKAP12*	intron	0.5329	0.00248404
rs16988201	*PTPRA*	intron	0.5324	0.00488301
rs1045580	*RPA3*	intron	0.5320	0.00270437
rs547976	*SFRS4*	intron	0.5308	0.00317025
rs684215	*PCCA*	intron	0.5302	0.00264730
rs9857526	*ADCY5*	intron	0.5294	0.00340934
rs458275	*PDE2A*	intron	0.5286	0.00278651
rs656024	*MGAT5B*	intron	0.5280	0.00279309
rs10493067	*SFPQ*	intron, phastCons element	0.5270	0.00281069
rs17080169	*RFC3*	intron	0.5267	0.00304077
rs1590345	*GSN*	intron	0.5261	0.00342262
rs532751	*RPS3*	intron	0.5257	0.00477382
rs3746692	*PTPRA*	intron	0.5256	0.00687662
rs12212415	*NUP43*	intron	0.5234	0.00874523
rs11775890	*EIF3S3*	intron	0.5216	0.00422599
rs9841477	*ADCY5*	intron	0.5208	0.00427301
rs2620386	*PLA2G4F*	intron	0.5196	0.00430490
rs11045355	*PDE3A*	intron	0.5167	0.00983470
rs555414	*VPS16*	intron	0.5166	0.00490839
rs2242107	*NR4A1*	intron, phastCons element	0.5161	0.00564583
rs341086	*PDE2A*	intron	0.5159	0.00571697
rs729916	*PDE1C*	intron	0.5156	0.00532810
rs7953019	*PTPRO*	intron	0.5152	0.00533911
rs9910060	*MGAT5B*	intron	0.5152	0.00533011
rs12019900	*PCCA*	intron	0.5151	0.00616792
rs6491545	*PCCA*	intron	0.5100	0.00578030
rs3115876	*CAPN3*	intron	0.5143	0.00619320
rs7257503	*IL4I1*	intron	0.5134	0.00719180
rs7818703	*SNTB1*	intron	0.5133	0.00603412
rs4787963	*GTF3C1*	intron	0.5133	0.00818659
rs596662	*PDE4B*	intron	0.5122	0.00729566
rs9442	*ITPR2*	intron	0.5121	0.00744518
rs2220168	*ITPR2*	intron	0.5121	0.00607126
rs2559825	*POLR3A*	intron	0.5113	0.00657438
rs6056505	*PLCB4*	intron	0.5107	0.00685158
rs11929107	*PLS1*	intron	0.5103	0.00841831

rs1888049	*RFC3*	intron		0.5074	0.00811002
rs2265520	*VAV2*	intron		0.5073	0.00781512
rs885219	*POMP*	intron		0.5072	0.00840051
rs9528255	*RFC3*	intron		0.5062	0.00876915
rs6037443	*PTPRA*	intron		0.5060	0.00848320
rs6664461	*CAPZB*	intron		0.5057	0.00946298
rs6977381	*ABP1*	intron		0.5053	0.00986581
rs6562150	*RFC3*	intron		0.5033	0.00997433
rs2512450	*MTDH*	intron		0.5031	0.00999221

[a]The aminoacid substitution is reported for nonsynonymous SNPs; SNPs annotated as "phastCons element" are located within non-coding genomic regions that display high sequence conservation among mammals (as described in the text).
