# Supplementary material for: Genome-Wide Identification of Susceptibility Alleles for Viral Infections through a Population Genetics Approach
Source: PLoS Genet. 2010 Feb 19;6(2):e1000849. doi: 10.1371/journal.pgen.1000849 (PMC2824813; doi:10.1371/journal.pgen.1000849)
Supplement: Table S4 — SNPs significantly associated with virus diversity. The table reports all SNPs that withstood Bonferroni correction at the genome-wide level (with α = 0.05) and displayed a Tau percentile rank higher than the 99th among MAF-matched SNPs, as described in the main text and in material and methods. SNPs are ranked according to the value of Tau. If the SNP is located within a genic region (or in the 500 upstream nucleotides) the gene symbol is reported. Also, the gene closest to the SNP and its distance (in bp) are indicated. The aminoacid substitution is reported for nonsynonymous variants; SNPs annotated as “phastCons element” are located within non-coding genomic regions that display high sequence conservation among mammals (as described in the text). (0.21 MB DOC) [file pgen.1000849.s004.doc]

**Table S4. SNP significantly associated with virus diversity.**

The table reports all SNP that withstood Bonferroni correction at the genome-wide level (with α = 0.05) and displayed a τ percentile rank higher than the 99^th^ among MAF-matched SNPs, as described in the main text and in material and methods. SNP are ranked according to the value of τ. If the SNP is located within a genic region (or in the 500 upstream nucleotides) the gene symbol is reported. Also, the gene closest to the SNP and its distance (in bp) are indicated. The aminoacid substitution is reported for nonsynonymous variants; SNPs annotated as "phastCons element" are located within non-coding genomic regions that display high sequence conservation among mammals (as described in the text).

| **SNP** | **Gene** | **Annotation** | **Closest gene** | **Distance (bp)** | **τ** |
| --- | --- | --- | --- | --- | --- |
| rs2359942 |  | intergenic | *PRDM2* | 297561 | 0.6321 |
| rs6981930 |  | intergenic | *TRHR* | 67787 | 0.6317 |
| rs12140284 |  | intergenic | *PPAP2B* | 347810 | 0.6307 |
| rs11793717 |  | intergenic | *CCRK* | 189534 | 0.6294 |
| rs10511316 | *CCDC80* | intron | *SLC35A5* | 44076 | 0.6270 |
| rs1135029 | *PDE2A* | A867A | *CENTD2* | 106822 | 0.6146 |
| rs7817222 |  | intergenic | *TRHR* | 17788 | 0.6125 |
| rs10254034 |  | intergenic | *MAGI2* | 148734 | 0.6104 |
| rs1011051 | *MYO5C* | intron | *GNB5* | 19692 | 0.6090 |
| rs993715 | *CNTNAP2* | intron |  |  | 0.6085 |
| rs7006331 |  | intergenic | *ZHX2* | 359350 | 0.6085 |
| rs11235559 | *PDE2A* | intron | *CENTD2* | 22076 | 0.6083 |
| rs2189883 | *CNTNAP2* | intron |  |  | 0.6081 |
| rs11581 | *KIAA1529* | Q1642Q | *TDRD7* | 35140 | 0.6070 |
| rs12202018 |  | intergenic | *HTR1B* | 253042 | 0.6067 |
| rs3785415 | *CDH15* | intron | *ZNF778* | 23547 | 0.6032 |
| rs4236799 |  | intergenic | *TRHR* | 23693 | 0.6027 |
| rs11601498 |  | intergenic | *OR52B2* | 7132 | 0.6014 |
| rs17256082 | *SCRN3* | intron | *GPR155* | 4010 | 0.6001 |
| rs647619 |  | intergenic | *ACTL8* | 29866 | 0.5995 |
| rs12576777 |  | intergenic | *SOX6* | 113903 | 0.5984 |
| rs189332 | *PDE2A* | intron | *CENTD2* | 15861 | 0.5984 |
| rs7768952 |  | intergenic | *RLBP1L2* | 1000 | 0.5979 |
| rs4852988 | *ANXA4* | intron | *GMCL1* | 78527 | 0.5966 |
| rs4575989 | *C1QTNF7* | intron | *CC2D2A* | 78929 | 0.5965 |
| rs12804437 |  | intergenic | *OR52B2* | 7441 | 0.5962 |
| rs7637370 | *CLDN18* | intron | *DZIP1L* | 44362 | 0.5960 |
| rs4786739 |  | intergenic | *A2BP1* | 394087 | 0.5959 |
| rs519332 | *EYA4* | intron | *RPS12* | 432569 | 0.5958 |
| rs4629443 | *C1QTNF7* | intron | *CC2D2A* | 85622 | 0.5955 |
| rs2188172 | *LHFPL3* | intron | *ORC5L* | 280423 | 0.5948 |
| rs10521211 |  | intergenic | *ELAC2* | 207784 | 0.5946 |
| rs1650893 | *LOC51149* | Q42R | *TBC1D9B* | 8691 | 0.5942 |
| rs2276209 |  | intergenic | *BRUNOL4* | 1796457 | 0.5937 |
| rs1322633 | *IBRDC1/RNF217* | intron | *TPD52L1* | 71599 | 0.5935 |
| rs7927476 | *NELL1* | intron | *SLC6A5* | 194126 | 0.5934 |
| rs2615666 | *TMEM132B* | intron |  |  | 0.5932 |
| rs569833 |  | intergenic | *EYA4* | 34559 | 0.5921 |
| rs4489081 |  | intergenic | *MAT2B* | 844776 | 0.5918 |
| rs548442 |  | intergenic | *C1orf100* | 82789 | 0.5911 |
| rs12534777 |  | intergenic | *MAGI2* | 138817 | 0.5910 |
| rs233992 |  | intergenic | *TACR3* | 16097 | 0.5909 |
| rs2278295 | *MYO5C* | intron | *GNB5* | 20577 | 0.5906 |
| rs1693569 |  | intergenic | *SNX31* | 15881 | 0.5896 |
| rs2339654 |  | intergenic | *RUNX1T1* | 496882 | 0.5894 |
| rs13020779 | *DIS3L2* | intron | *ALPP* | 93747 | 0.5893 |
| rs10861946 |  | intergenic | *ISCU* | 5971 | 0.5893 |
| rs1719596 | *LEPREL1* | intron | *TP63* | 97673 | 0.5893 |
| rs12328023 |  | intergenic | *ASB3* | 335542 | 0.5893 |
| rs1065154 | *SQSTM1/LOC51149/*  *C5orf45* | 3' UTR / intron | *TBC1D9B* | 24155 | 0.5893 |
| rs12145973 | *IL19* | intron | *IL19* | 7496 | 0.5890 |
| rs4889551 |  | intergenic | *ZNF267* | 158632 | 0.5885 |
| rs1890139 | *PCCA* | intron | *ZIC2* | 143073 | 0.5883 |
| rs10929675 |  | intergenic | *NOL10* | 5254 | 0.5880 |
| rs2146136 |  | intergenic | *KIAA0774* | 54061 | 0.5879 |
| rs6505045 | *ANKFN1* | intron | *PCTP* | 403610 | 0.5874 |
| rs2187942 |  | intergenic | *PRDM2* | 307566 | 0.5872 |
| rs4953260 | *PRKCE* | intron | *SRBD1* | 195316 | 0.5872 |
| rs9865950 |  | intergenic | *ROBO1* | 634259 | 0.5870 |
| rs4077341 | *TNFRSF10C* | intron | *TNFRSF10D* | 30755 | 0.5869 |
| rs7175118 |  | intergenic, phastCons element | *UNC13C* | 35261 | 0.5867 |
| rs2793434 | *GPLD1* | intron | *GPLD1* | 12646 | 0.5866 |
| rs6740690 |  | intergenic | *MGAT5* | 233794 | 0.5860 |
| rs6743570 |  | intergenic | *MGAT5* | 233699 | 0.5860 |
| rs717538 |  | intergenic | *PRDM2* | 305655 | 0.5856 |
| rs9314015 |  | intergenic | *MAT2B* | 836057 | 0.5853 |
| rs11760238 | *LHFPL3* | intron | *ORC5L* | 275082 | 0.5850 |
| rs6507652 |  | intergenic | *KIAA1632* | 2756 | 0.5849 |
| rs11138465 |  | intergenic | *TLE4* | 320931 | 0.5846 |
| rs2398121 |  | intergenic | *CUGBP2* | 869925 | 0.5846 |
| rs1155422 |  | intergenic | *MAP9* | 110551 | 0.5843 |
| rs6599300 | *MAEA* | intron | *KIAA1530* | 35834 | 0.5843 |
| rs13340461 | *CCND3* | intron |  |  | 0.5840 |
| rs7614718 |  | intergenic | *ROBO1* | 759393 | 0.5838 |
| rs6803290 |  | intergenic | *FHIT* | 42867 | 0.5838 |
| rs11784487 | *ANK1* | intron | *ANK1* | 79584 | 0.5835 |
| rs10849446 | *SCNN1A* | intron | *LTBR* | 14064 | 0.5831 |
| rs12186418 | *PDZD2* | intron | *GOLPH3* | 226233 | 0.5831 |
| rs1517974 |  | intergenic | *NXPH1* | 1031626 | 0.5829 |
| rs640239 |  | intergenic | *C1orf100* | 80258 | 0.5826 |
| rs4698103 | *C1QTNF7* | intron | *CC2D2A* | 92216 | 0.5825 |
| rs12940715 |  | intergenic | *CDC42EP4* | 169588 | 0.5824 |
| rs11710972 |  | intergenic | *CCDC66* | 37867 | 0.5823 |
| rs6752715 |  | intergenic |  |  | 0.5822 |
| rs9929377 |  | intergenic | *DYNLRB2* | 165018 | 0.5820 |
| rs9825550 |  | intergenic, phastCons element | *ROBO1* | 671198 | 0.5820 |
| rs9955285 |  | intergenic | *KIAA1632* | 1813 | 0.5817 |
| rs2484124 |  | intergenic | *C1orf100* | 88934 | 0.5815 |
| rs17282579 | *CLDN18* | Near 5' gene/ intron | *CLDN18* | 138 | 0.5806 |
| rs4932557 |  | intergenic | *SLCO3A1* | 278682 | 0.5805 |
| rs2600306 | *CNTN4* | intron | *CNTN4* | 270163 | 0.5800 |
| rs11581254 |  | intergenic | *FAM78B* | 16107 | 0.5798 |
| rs2825726 |  | intergenic | *PRSS7* | 1267199 | 0.5798 |
| rs4259254 |  | intergenic | *NOX3* | 401097 | 0.5796 |
| rs3825877 |  | intergenic | *ZSCAN2* | 7684 | 0.5795 |
| rs2292463 |  | intergenic | *ZSCAN2* | 8803 | 0.5795 |
| rs11665332 |  | intergenic | *C18orf62* | 101085 | 0.5795 |
| rs7340104 | *LZTR2* | intron |  |  | 0.5794 |
| rs6026302 | *APCDD1L* | intron | *VAPB* | 39169 | 0.5793 |
| rs6432127 | *NOL10* | intron | *ATP6V1C2* | 104629 | 0.5792 |
| rs2142569 |  | intergenic | *ACTRT2* | 5060 | 0.5790 |
| rs7746067 | *KCNQ5* | intron | *RIMS1* | 247590 | 0.5787 |
| rs10753700 |  | intergenic | *FAM78B* | 27589 | 0.5782 |
| rs11135310 |  | intergenic | *MAT2B* | 840625 | 0.5781 |
| rs867426 |  | intergenic | *RGS18* | 579597 | 0.5778 |
| rs6718438 | *DIS3L2* | intron, phastCons element | *ALPP* | 87781 | 0.5778 |
| rs705308 |  | intergenic | *LMTK2* | 40833 | 0.5778 |
| rs700550 | *LRP2* | intron | *BBS5* | 177549 | 0.5777 |
| rs971403 | *LAMA4* | intron | *C6orf225* | 64008 | 0.5776 |
| rs9989425 | *XYLT1* | intron | *LOC339047* | 798014 | 0.5776 |
| rs482522 |  | intergenic | *SPPL3* | 6172 | 0.5774 |
| rs12371477 | *GAS2L3* | intron | *NR1H4* | 56666 | 0.5773 |
| rs2659501 | *PPP3CA* | intron | *BANK1* | 617956 | 0.5771 |
| rs6996401 |  | intergenic | *ZHX2* | 445662 | 0.5768 |
| rs2282529 |  | intergenic | *NPAS4* | 4334 | 0.5760 |
| rs4671675 | *FLJ16124* | intron | *SPRED2* | 201999 | 0.5758 |
| rs716527 | *FMN2* | intron | *GREM2* | 225717 | 0.5757 |
| rs10491196 |  | intergenic | *ANKFN1* | 15025 | 0.5755 |
| rs1363347 |  | intergenic | *CACNA1A* | 6275 | 0.5754 |
| rs10898866 | *CENTD2* | intron | *PDE2A* | 25687 | 0.5753 |
| rs3924658 |  | intergenic | *SH3BP4* | 103737 | 0.5752 |
| rs1986656 | *TMEM110* | intron | *SFMBT1* | 25223 | 0.5747 |
| rs6760896 | *DIS3L2* | intron | *ALPP* | 70424 | 0.5747 |
| rs13234705 | *LHFPL3* | intron, phastCons element | *ORC5L* | 283847 | 0.5746 |
| rs3732114 | *NOL10* | L662L | *ODC1* | 123825 | 0.5744 |
| rs7259736 |  | intergenic | *LOC284402* | 10774 | 0.5743 |
| rs7074891 | *TRDMT1* | 3' UTR | *CUBN* | 16658 | 0.5741 |
| rs2201057 |  | intergenic | *ERC2* | 31011 | 0.5739 |
| rs2016977 | *ST8SIA1* | intron | *KIAA0528* | 171633 | 0.5738 |
| rs3782525 | *ST8SIA1* | intron | *KIAA0528* | 163435 | 0.5738 |
| rs7935223 | *GALNTL4* | intron, phastCons element | *USP47* | 266635 | 0.5738 |
| rs11854089 |  | intergenic | *MEIS2* | 99084 | 0.5738 |
| rs377962 | *EYA4* | intron | *RPS12* | 528201 | 0.5737 |
| rs942576 | *COL13A1* | intron | *H2AFY2* | 149573 | 0.5731 |
| rs4130023 | *CCND3* | intron, phastCons element |  |  | 0.5725 |
| rs7287616 |  | intergenic | *IGLL1* | 7357 | 0.5725 |
| rs1346690 | *NELL1* | intron | *SLC6A5* | 194633 | 0.5724 |
| rs2594935 | *OCA2* | intron | *HERC2* | 171149 | 0.5724 |
| rs5937220 |  | intergenic | *FAM155B* | 84560 | 0.5723 |
| rs2237315 | *DFNA5* | intron | *MPP6* | 34352 | 0.5722 |
| rs10771234 |  | intergenic | *IFLTD1* | 158546 | 0.5722 |
| rs12828155 | *OVCH1* | intron | *ERGIC2* | 51812 | 0.5722 |
| rs4800476 | *C18orf45* | intron | *RIOK3* | 36385 | 0.5721 |
| rs10891727 |  | intergenic | *FAM55D* | 45531 | 0.5720 |
| rs2527049 | *CNTNAP2* | intron |  |  | 0.5719 |
| rs1038511 |  | intergenic | *MGAT5* | 221553 | 0.5719 |
| rs5917027 | *CLDN2* | intron |  |  | 0.5718 |
| rs11150843 | *GAA* | R223H |  |  | 0.5717 |
| rs8112457 |  | intergenic | *ZNF302* | 32646 | 0.5715 |
| rs4666014 | *RBKS* | intron, phastCons element | *MRPL33* | 16567 | 0.5712 |
| rs16957806 |  | intergenic | *CDH13* | 34819 | 0.5712 |
| rs1874108 | *FREM1* | intron, phastCons element | *CER1* | 53064 | 0.5711 |
| rs4677735 | *CCDC50* | intron | *UTS2D* | 64488 | 0.5710 |
| rs2345043 | *LPHN3* | intron |  |  | 0.5705 |
| rs2888312 | *TMEM16K* | intron | *SNRK* | 92333 | 0.5705 |
| rs1055636 | *SLFN5* | 3' UTR | *AMAC1* | 72943 | 0.5705 |
| rs2714174 | *LRP1B* | intron | *KYNU* | 1516193 | 0.5702 |
| rs3123171 |  | intergenic | *TCERG1L* | 110205 | 0.5701 |
| rs294278 |  | intergenic | *GADL1* | 191758 | 0.5701 |
| rs341602 |  | intergenic | *C2orf34* | 65774 | 0.5699 |
| rs12554965 |  | intergenic | *PGM5* | 10464 | 0.5698 |
| rs11746351 |  | intergenic, phastCons element | *MAT2B* | 1361025 | 0.5698 |
| rs822094 | *SORCS1* | intron | *SORCS3* | 1693390 | 0.5697 |
| rs10840658 |  | intergenic | *LMO3* | 521664 | 0.5697 |
| rs1859458 | *TMEM132B* | intron |  |  | 0.5696 |
| rs12712519 | *VIT* | intron | *STRN* | 105974 | 0.5696 |
| rs7076053 |  | intergenic | *PCBD1* | 143947 | 0.5695 |
| rs329134 |  | intergenic | *CLDN18* | 2059 | 0.5694 |
| rs3741604 | *HELB* | R9R | *GRIP1* | 46388 | 0.5692 |
| rs11934405 |  | intergenic | *KIAA1712* | 29322 | 0.5692 |
| rs933561 |  | intergenic | *ZNF423* | 13758 | 0.5691 |
| rs873711 | *VPS16* | intron | *FAM113A* | 6136 | 0.5689 |
| rs2825730 |  | intergenic | *PRSS7* | 1272068 | 0.5688 |
| rs7305924 | *BCAT1* | intron | *LOC196415* | 79757 | 0.5687 |
| rs9851577 |  | intergenic | *ALDH1L1* | 8825 | 0.5687 |
| rs1402470 | *LRP1B* | intron | *KYNU* | 1515792 | 0.5685 |
| rs2327960 | *C20orf133* | intron | *KIF16B* | 557981 | 0.5684 |
| rs2550904 | *CHST6* | intron | *TMEM170A* | 29927 | 0.5683 |
| rs1922889 | *CNTNAP2* | intron |  |  | 0.5682 |
| rs1463184 |  | intergenic | *RUNX1T1* | 505771 | 0.5681 |
| rs7495265 |  | intergenic | *SLCO3A1* | 246030 | 0.5681 |
| rs2289599 |  | intergenic | *AP3B1* | 90000 | 0.5679 |
| rs12929785 |  | intergenic | *C16orf68* | 361803 | 0.5678 |
| rs5752178 |  | intergenic | *ADRBK2* | 15034 | 0.5677 |
| rs7253017 |  | intergenic | *GPR32* | 5828 | 0.5677 |
| rs12540386 |  | intergenic | *MAGI2* | 181581 | 0.5675 |
| rs1894599 |  | intergenic | *CYTH4* | 5249 | 0.5675 |
| rs272817 |  | intergenic | *MAP7D1* | 50 | 0.5675 |
| rs10517729 |  | intergenic | *FSTL5* | 290770 | 0.5673 |
| rs2552241 |  | intergenic | *MCPH1* | 410236 | 0.5673 |
| rs2793675 |  | intergenic | *PPAP2B* | 202464 | 0.5671 |
| rs1014936 |  | intergenic | *TDRD3* | 133232 | 0.5669 |
| rs2811715 |  | intergenic | *CDKN2A* | 37179 | 0.5668 |
| rs7287117 |  | intergenic | *PARVG* | 201 | 0.5667 |
| rs2299546 | *GRM8* | intron | *ZNF800* | 182482 | 0.5664 |
| rs2025324 | *JMJD2C* | intron | *GLDC* | 456697 | 0.5661 |
| rs11242715 | *GMDS* | intron | *FOXC1* | 75870 | 0.5661 |
| rs11071869 | *MEGF11* | intron | *DIS3L* | 95978 | 0.5660 |
| rs7712010 | *PDZD2* | intron | *GOLPH3* | 216936 | 0.5659 |
| rs2045806 | *C8orf47* | intron | *HRSP12* | 23463 | 0.5659 |
| rs12336938 |  | intergenic | *PALM2* | 81552 | 0.5658 |
| rs17008470 |  | intergenic | *ZNF385D* | 80156 | 0.5656 |
| rs17376891 |  | intergenic | *MAT2B* | 1306192 | 0.5655 |
| rs17656058 | *CLEC4F* | intron, phastCons element | *CD207* | 18781 | 0.5655 |
| rs2077759 |  | intergenic | *GNAI1* | 39785 | 0.5655 |
| rs1369093 | *ADAMTS3* | intron | *NPFFR2* | 231413 | 0.5654 |
| rs4932583 | *SLCO3A1* | intron | *ST8SIA2* | 508050 | 0.5653 |
| rs6725950 | *FLJ16124* | intron | *SPRED2* | 204607 | 0.5652 |
| rs6546171 | *FLJ16124* | intron, phastCons element | *SPRED2* | 223878 | 0.5651 |
| rs3923120 |  | intergenic | *PTPRD* | 87346 | 0.5651 |
| rs7129085 | *INCENP* | E644D | *SCGB1D1* | 44513 | 0.5651 |
| rs480807 |  | intergenic | *AJAP1* | 459520 | 0.5651 |
| rs2633604 |  | intergenic | *CD200* | 38404 | 0.5650 |
| rs3809254 | *PARP11* | intron | *EFCAB4B* | 79003 | 0.5650 |
| rs9946933 | *KIAA1632* | intron | *PSTPIP2* | 50239 | 0.5650 |
| rs1431486 | *PNLIPRP3* | intron | *C10orf96* | 53591 | 0.5650 |
| rs942151 |  | intergenic |  |  | 0.5648 |
| rs708228 | *CTNND1* | 3' UTR, phastCons element | *C11orf31* | 74779 | 0.5647 |
| rs8023445 | *SHC4* | intron | *EID1* | 20411 | 0.5646 |
| rs13182372 | *PDZD2* | intron | *GOLPH3* | 224829 | 0.5646 |
| rs989251 |  | intergenic, phastCons element | *ZNF302* | 28988 | 0.5644 |
| rs4698374 | *C1QTNF7* | intron | *CC2D2A* | 96802 | 0.5642 |
| rs4679354 |  | intergenic | *C3orf56* | 30678 | 0.5640 |
| rs4800158 | *C18orf45* | intron | *RIOK3* | 46998 | 0.5640 |
| rs4776759 | *MEGF11* | intron | *DIS3L* | 96121 | 0.5639 |
| rs7151279 |  | intergenic | *C14orf180* | 138782 | 0.5638 |
| rs7591064 |  | intergenic | *FSHR* | 12050 | 0.5636 |
| rs1032429 |  | intergenic | *KCNA2* | 3222 | 0.5636 |
| rs11096686 | *FAM49A* | intron | *MYCN* | 674793 | 0.5636 |
| rs11079764 |  | intergenic | *MYL4* | 8648 | 0.5635 |
| rs9300574 |  | intergenic | *CLYBL* | 30269 | 0.5634 |
| rs10520891 |  | intergenic | *PRDM9* | 49434 | 0.5633 |
| rs1859455 | *TMEM132B* | intron |  |  | 0.5633 |
| rs7594352 |  | intergenic |  |  | 0.5632 |
| rs12111265 | *KCNQ5* | intron | *RIMS1* | 257231 | 0.5632 |
| rs6591555 |  | intergenic | *PLAC1L* | 62054 | 0.5632 |
| rs2683824 | *LRP1B* | intron | *KYNU* | 1526197 | 0.5629 |
| rs2795610 |  | intergenic | *KIAA1462* | 59539 | 0.5628 |
| rs10762485 |  | intergenic | *PSAP* | 8346 | 0.5628 |
| rs2196961 |  | intergenic | *SOX6* | 91970 | 0.5627 |
| rs13160298 |  | intergenic | *EBF1* | 326605 | 0.5626 |
| rs2346115 |  | intergenic | *TMEM132B* | 321029 | 0.5625 |
| rs1316379 |  | intergenic | *EBF1* | 233531 | 0.5625 |
| rs13082005 | *ATG3* | intron | *SLC35A5* | 10037 | 0.5625 |
| rs7916768 |  | intergenic | *PCDH15* | 413069 | 0.5624 |
| rs1958313 |  | intergenic | *DHRS2* | 132862 | 0.5623 |
| rs10793036 | *PDE2A* | intron | *CENTD2* | 73934 | 0.5619 |
| rs13028997 |  | intergenic | *NR4A2* | 20758 | 0.5618 |
| rs2546078 | *MEGF10* | intron | *PRRC1* | 184664 | 0.5617 |
| rs4549077 |  | intergenic | *SLC4A3* | 794070 | 0.5616 |
| rs7656744 |  | intergenic | *TACR3* | 60845 | 0.5616 |
| rs13236941 |  | intergenic | *PON1* | 1936 | 0.5616 |
| rs341399 | *RORA* | intron | *RORA* | 210313 | 0.5616 |
| rs13293826 |  | intergenic | *TUSC1* | 522602 | 0.5615 |
| rs6889488 |  | intergenic | *FAM173B* | 6890 | 0.5615 |
| rs3759324 |  | intergenic | *SCNN1A* | 946 | 0.5614 |
| rs10512396 |  | intergenic | *PALM2* | 57049 | 0.5613 |
| rs331617 |  | intergenic | *BMP7* | 103960 | 0.5613 |
| rs2047683 | *RIOK3* | intron | *C18orf45* | 31878 | 0.5612 |
| rs12034991 | *SYT14* | intron | *C1orf107* | 114390 | 0.5609 |
| rs11710107 | *TMEM16K* | intron | *SNRK* | 81280 | 0.5609 |
| rs6446675 |  | intergenic | *STX18* | 131521 | 0.5609 |
| rs12198275 |  | intergenic | *RLBP1L2* | 10680 | 0.5609 |
| rs1155624 |  | intergenic | *CENPC1* | 590945 | 0.5608 |
| rs225843 |  | intergenic | *PRKD1* | 155879 | 0.5607 |
| rs4751118 | *MGMT* | intron | *EBF3* | 90140 | 0.5606 |
| rs558275 |  | intergenic | *SPPL3* | 4143 | 0.5604 |
| rs756802 |  | intergenic | *USP31* | 63153 | 0.5604 |
| rs9872812 |  | intergenic | *GADL1* | 186174 | 0.5602 |
| rs10518938 | *THSD4* | intron | *LOC645296* | 87918 | 0.5601 |
| rs17172715 |  | intergenic | *IGFBP3* | 636851 | 0.5601 |
| rs6593185 | *GRB10* | intron | *GRB10* | 58595 | 0.5600 |
| rs1402517 |  | intergenic | *MDGA2* | 306536 | 0.5598 |
| rs677661 |  | intergenic | *PSMB2* | 26775 | 0.5595 |
| rs7755100 |  | intergenic | *MAN1A1* | 808080 | 0.5595 |
| rs2501254 | *HSPG2* | intron | *ELA3B* | 70940 | 0.5595 |
| rs11689619 | *VIT* | intron | *STRN* | 105759 | 0.5595 |
| rs9397336 |  | intergenic | *PLEKHG1* | 29836 | 0.5595 |
| rs12565323 |  | intergenic | *PRMT6* | 693336 | 0.5592 |
| rs9482421 |  | intergenic | *TRDN* | 31576 | 0.5591 |
| rs9543335 |  | intergenic | *KLF5* | 294333 | 0.5590 |
| rs9543336 |  | intergenic | *KLF5* | 296236 | 0.5590 |
| rs4287543 |  | intergenic | *SV2B* | 274855 | 0.5589 |
| rs10013604 |  | intergenic | *GRXCR1* | 568490 | 0.5588 |
| rs4896651 |  | intergenic | *AIG1* | 29952 | 0.5588 |
| rs916430 |  | intergenic | *PRDM2* | 294218 | 0.5587 |
| rs260368 |  | intergenic | *IGFBP3* | 502471 | 0.5587 |
| rs7782875 |  | intergenic | *MAGI2* | 140402 | 0.5587 |
| rs566160 |  | intergenic | *LIPC* | 85395 | 0.5586 |
| rs12612231 | *DIS3L2* | intron | *ALPP* | 67413 | 0.5585 |
| rs233990 |  | intergenic | *TACR3* | 26303 | 0.5585 |
| rs4332691 | *WDR72* | intron | *UNC13C* | 414738 | 0.5584 |
| rs11603266 |  | intergenic | *FAT3* | 1050339 | 0.5584 |
| rs4632107 |  | intergenic | *SLCO3A1* | 256766 | 0.5584 |
| rs4478120 |  | intergenic | *GADL1* | 169986 | 0.5583 |
| rs1016492 |  | intergenic | *TACR3* | 44937 | 0.5583 |
| rs1019947 |  | intergenic | *EMCN* | 8318 | 0.5582 |
| rs2125714 |  | intergenic | *ST8SIA4* | 443416 | 0.5582 |
| rs11211632 |  | intergenic, phastCons element | *SLC5A9* | 281943 | 0.5581 |
| rs10496358 |  | intergenic | *MAP4K4* | 26114 | 0.5580 |
| rs9730058 |  | intergenic | *DISC1* | 65907 | 0.5579 |
| rs4540401 | *GDAP1* | intron | *JPH1* | 31920 | 0.5579 |
| rs12085924 |  | intergenic | *PLD5* | 255271 | 0.5579 |
| rs9290505 |  | intergenic | *NAALADL2* | 278737 | 0.5577 |
| rs12053101 | *SCRN3* | intron | *GPR155* | 17471 | 0.5577 |
| rs17713587 |  | intergenic | *MSX1* | 144870 | 0.5576 |
| rs886001 | *GRM8* | intron | *ZNF800* | 172890 | 0.5575 |
| rs1896799 | *SH3GL3* | intron | *ADAMTSL3* | 74986 | 0.5575 |
| rs12568035 | *LAMB3* | intron | *CAMK1G* | 16499 | 0.5575 |
| rs9908079 | *SMTNL2* | intron | *GGT6* | 33208 | 0.5574 |
| rs1011870 |  | intergenic | *ERGIC2* | 23025 | 0.5573 |
| rs9866516 |  | intergenic | *ROBO1* | 664907 | 0.5573 |
| rs1157180 | *EMCN* | intron | *DDIT4L* | 257048 | 0.5572 |
| rs12042893 |  | intergenic | *FCRLA* | 1583 | 0.5572 |
| rs2275254 | *CHIA* | F354S | *C1orf88* | 27220 | 0.5571 |
| rs11662508 |  | intergenic | *RBBP8* | 46074 | 0.5571 |
| rs3732941 |  | intergenic | *ATG7* | 9526 | 0.5569 |
| rs11745765 |  | intergenic | *PRDM9* | 31321 | 0.5566 |
| rs3856982 | *MAEA* | intron | *KIAA1530* | 12240 | 0.5565 |
| rs9290933 |  | intergenic | *IL1RAP* | 18014 | 0.5565 |
| rs2850350 | *PPP3CA* | intron | *BANK1* | 506378 | 0.5564 |
| rs939856 | *SOX5* | intron | *C12orf67* | 221332 | 0.5563 |
| rs1848116 | *PPP3CA* | intron | *BANK1* | 607823 | 0.5563 |
| rs1071664 | *TK1* | P11P | *AFMID* | 393 | 0.5563 |
| rs7930102 | *OR9Q1* | intron | *OR6Q1* | 15381 | 0.5562 |
| rs1041205 |  | intergenic | *OLFML2A* | 3845 | 0.5560 |
| rs11807256 |  | intergenic | *RTCD1* | 12437 | 0.5559 |
| rs7736502 | *PDZD2* | intron | *GOLPH3* | 224451 | 0.5559 |
| rs7107376 | *FCHSD2* | intron | *ATG16L2* | 100311 | 0.5558 |
| rs2195139 |  | intergenic | *DKFZp564N2472* | 172465 | 0.5557 |
| rs5014235 |  | intergenic | *AP3B1* | 88488 | 0.5556 |
| rs6770985 |  | intergenic | *CADPS* | 145529 | 0.5554 |
| rs2607061 |  | intergenic | *CDH17* | 15316 | 0.5553 |
| rs9563673 | *STARD13* | intron | *RFC3* | 152679 | 0.5551 |
| rs7940652 |  | intergenic | *NCAM1* | 288183 | 0.5551 |
| rs2707575 | *CNTNAP2* | intron |  |  | 0.5550 |
| rs677344 | *VPS16, PTPRA* | intron, intron | *PTPRA* | 7852 | 0.555 |
| rs13235067 |  | intergenic | *LOC154907* | 809320 | 0.5549 |
| rs1500127 |  | intergenic | *ODZ2* | 971860 | 0.5548 |
| rs7201128 |  | intergenic | *SNX29* | 37784 | 0.5548 |
| rs2312547 |  | intergenic | *ANXA4* | 29285 | 0.5547 |
| rs2193776 | *FAM49A* | intron | *MYCN* | 676838 | 0.5546 |
| rs4290027 |  | intergenic | *POU3F1* | 372079 | 0.5546 |
| rs2623630 | *CSMD1* | intron | *MYOM2* | 1425614 | 0.5544 |
| rs2594934 | *OCA2* | intron | *HERC2* | 173472 | 0.5544 |
| rs2850976 | *PPP3CA* | intron | *BANK1* | 509478 | 0.5544 |
| rs4777882 |  | intergenic | *RGMA* | 351768 | 0.5543 |
| rs2192973 | *GRIN2B* | intron | *C12orf36* | 366910 | 0.5542 |
| rs4776758 | *MEGF11* | intron | *DIS3L* | 111615 | 0.5540 |
| rs1138803 | *GMDS* | intron | *FOXC1* | 82328 | 0.5540 |
| rs1399796 |  | intergenic | *NAALADL2* | 280651 | 0.5538 |
| rs356874 |  | intergenic | *CDH2* | 541041 | 0.5537 |
| rs929707 | *ATP2B2* | intron | *SEC13* | 114171 | 0.5536 |
| rs13006813 | *VIT* | intron | *STRN* | 106135 | 0.5536 |
| rs12764801 |  | intergenic | *ASB13* | 3754 | 0.5533 |
| rs10786777 | *NRG3* | intron | *SH2D4B* | 1289309 | 0.5533 |
| rs953035 | *PSMB2* | intron | *TFAP2E* | 18581 | 0.5532 |
| rs6439858 |  | intergenic | *RBP2* | 21142 | 0.5530 |
| rs6465657 | *LMTK2* | intron | *BHLHB8* | 25238 | 0.5528 |
| rs1909340 | *LEMD3* | intron | *MSRB3* | 63804 | 0.5528 |
| rs10401065 |  | intergenic |  |  | 0.5528 |
| rs12415467 | *PTPRE* | intron | *CLRN3* | 53254 | 0.5528 |
| rs980618 | *IL16* | intron | *IL16* | 69257 | 0.5524 |
| rs2063413 |  | intergenic, phastCons element | *STX18* | 110582 | 0.5522 |
| rs991250 | *PTPRD* | intron |  |  | 0.5522 |
| rs9483335 | *ENPP3* | intron | *OR2A4* | 36533 | 0.5521 |
| rs4622605 | *LOC284274* | intron | *TSHZ1* | 137107 | 0.5520 |
| rs2541886 | *UNG* | intron | *ALKBH2* | 6319 | 0.5519 |
| rs8142159 |  | intergenic | *TTLL1* | 5205 | 0.5518 |
| rs9850224 |  | intergenic | *SLC25A36* | 85374 | 0.5517 |
| rs1417144 |  | intergenic | *PRDM2* | 314510 | 0.5517 |
| rs7760979 | *KCNQ5* | intron | *RIMS1* | 290770 | 0.5516 |
| rs6957435 | *GRM8* | intron | *ZNF800* | 169263 | 0.5516 |
| rs168206 | *DLGAP1* | intron | *TGIF1* | 235366 | 0.5516 |
| rs1289407 |  | intergenic |  |  | 0.5516 |
| rs7977896 |  | intergenic | *OVCH1* | 21492 | 0.5514 |
| rs2369535 |  | intergenic | *USP31* | 64199 | 0.5514 |
| rs1057804 | *GNG2* | 3' UTR, phastCons element | *C14orf166* | 22172 | 0.5514 |
| rs11110445 |  | intergenic | *GAS2L3* | 3453 | 0.5512 |
| rs6733161 | *NOL10* | intron | *ATP6V1C2* | 93176 | 0.5512 |
| rs2345016 |  | intergenic | *IGFBP3* | 530313 | 0.5511 |
| rs10409243 |  | intergenic | *DNMT1* | 27233 | 0.5510 |
| rs1931725 |  | intergenic | *DOCK1* | 17747 | 0.5510 |
| rs4425734 |  | intergenic | *SLC7A13* | 34971 | 0.5509 |
| rs7642758 |  | intergenic | *GADL1* | 171549 | 0.5508 |
| rs10857274 | *DCHS2* | intron | *PLRG1* | 150235 | 0.5506 |
| rs4386 | *TOM1* | intron | *HMOX1* | 35915 | 0.5506 |
| rs2525570 | *NF1* | intron | *EVI2A* | 32528 | 0.5506 |
| rs2057367 | *SAMD4A* | intron | *GCH1* | 103632 | 0.5505 |
| rs2290652 | *ZNF302* | Y89C | *ZNF181* | 49884 | 0.5504 |
| rs1011489 |  | intergenic | *A2BP1* | 401738 | 0.5504 |
| rs6709076 | *IQCA* | intron | *CXCR7* | 72690 | 0.5503 |
| rs4814097 |  | intergenic | *BTBD3* | 451177 | 0.5502 |
| rs346860 |  | intergenic | *ST8SIA4* | 479957 | 0.5499 |
| rs9652853 |  | intergenic | *MSI2* | 52339 | 0.5498 |
| rs7695691 | *MAEA* | intron | *KIAA1530* | 36471 | 0.5496 |
| rs1876201 |  | intergenic | *COL22A1* | 56761 | 0.5496 |
| rs13301412 |  | intergenic | *ASS1* | 11571 | 0.5495 |
| rs1376307 | *LPHN3* | intron |  |  | 0.5494 |
| rs7643541 |  | intergenic | *NAALADL2* | 220134 | 0.5494 |
| rs9855684 |  | intergenic | *NAALADL2* | 275859 | 0.5492 |
| rs1570269 |  | intergenic | *HABP2* | 25575 | 0.5492 |
| rs595961 | *EIF2C1* | intron, phastCons element | *EIF2C3* | 28991 | 0.5491 |
| rs4943130 | *STARD13* | intron | *RFC3* | 155489 | 0.5490 |
| rs565280 | *SQSTM1* | intron | *C5orf45* | 10339 | 0.5490 |
| rs10910323 | *SLC35F3* | intron | *KCNK1* | 319335 | 0.5489 |
| rs2580312 |  | intergenic | *CDYL2* | 3334 | 0.5488 |
| rs12604430 | *KIAA1632* | intron | *PSTPIP2* | 37690 | 0.5487 |
| rs2275603 | *FCRLA* | S203G | *FCRLB* | 10676 | 0.5484 |
| rs11097328 |  | intergenic | *GRID2* | 533910 | 0.5483 |
| rs9423593 |  | intergenic | *AKR1C4* | 71437 | 0.5482 |
| rs10518680 | *MEGF11* | intron | *DIS3L* | 62475 | 0.5482 |
| rs1150903 |  | intergenic | *ACTL8* | 118190 | 0.5481 |
| rs4506180 |  | intergenic | *TRHR* | 13907 | 0.5480 |
| rs12552010 |  | intergenic | *WDR38* | 3355 | 0.5479 |
| rs806739 |  | intergenic | *PMFBP1* | 231717 | 0.5479 |
| rs9442831 | *KCNQ5* | intron | *RIMS1* | 248125 | 0.5477 |
| rs372454 | *MMD2* | 3' UTR | *RADIL* | 22840 | 0.5477 |
| rs2966849 |  | intergenic | *FAM92B* | 37568 | 0.5475 |
| rs9374007 |  | intergenic | *OSTM1* | 4284 | 0.5473 |
| rs7121400 | *NELL1* | intron | *SLC6A5* | 197828 | 0.5473 |
| rs431736 |  | intergenic | *CADM1* | 532192 | 0.5473 |
| rs6828071 | *FLJ12993* | intron | *ENOPH1* | 49546 | 0.5473 |
| rs1107179 | *NMB* | 3' UTR | *WDR73* | 841 | 0.5471 |
| rs17315339 |  | intergenic | *CTSO* | 255241 | 0.5471 |
| rs4805985 |  | intergenic | *LSM14A* | 67354 | 0.5469 |
| rs11071503 | *CHRNA7* | intron | *OTUD7A* | 387414 | 0.5467 |
| rs7954956 | *FLJ21963* | intron | *LIN7A* | 147625 | 0.5465 |
| rs4342312 |  | intergenic, phastCons element | *NR2F1* | 441152 | 0.5463 |
| rs9565069 | *NBEA* | intron | *MAB21L1* | 304215 | 0.5459 |
| rs11155550 |  | intergenic | *SASH1* | 231418 | 0.5459 |
| rs332040 | *MFHAS1* | intron | *THEX1* | 129825 | 0.5457 |
| rs341087 | *PDE2A* | intron | *CENTD2* | 79629 | 0.5457 |
| rs4888116 |  | intergenic | *C16orf61* | 51256 | 0.5455 |
| rs296422 | *EYA4* | intron | *TCF21* | 484500 | 0.5455 |
| rs1957358 | *SAMD4A* | intron | *GCH1* | 86248 | 0.5451 |
| rs7797420 |  | intergenic | *ATP6V1F* | 9944 | 0.5449 |
| rs1605527 |  | intergenic | *ZNF385D* | 85796 | 0.5447 |
| rs3784929 | *KARS* | intron | *TERF2IP* | 4656 | 0.5447 |
| rs6472619 |  | intergenic | *MSC* | 43266 | 0.5447 |
| rs29824 |  | intergenic | *GLRA1* | 210553 | 0.5445 |
