# Supplementary material for: Genome-Wide Identification of Susceptibility Alleles for Viral Infections through a Population Genetics Approach
Source: PLoS Genet. 2010 Feb 19;6(2):e1000849. doi: 10.1371/journal.pgen.1000849 (PMC2824813; doi:10.1371/journal.pgen.1000849)
Supplement: Table S5 — Correlations between SNPs associated with virus diversity and other climatic variables. The table shows correlation coefficients between each SNP associated with virus diversity and the following climatic variables: average annual maximum temperature (Tmax), average annual minimum temperature (Tmin), short wave radiation flux (Irradiation SW). After Bonferroni correction all p values were >0.05. (0.42 MB DOC) [file pgen.1000849.s005.doc]

**Table S5. Correlations between SNPs associated with virus diversity and other climatic variables.**

The table shows correlation coefficients between each SNP associated with virus diversity and the following climatic variables: average annual maximum temperature (Tmax), average annual minimum temperature (Tmin), short wave radiation flux (Irradiation SW). After Bonferroni correction all *p* values were > 0.05.

|  | **Tmin** | | **Tmax** | | **Irradiation SW** | |
| --- | --- | --- | --- | --- | --- | --- |
|  | **τ** | ***p* value** | **τ** | ***p* value** | **τ** | ***p* value** |
| rs2142569 | 0.0849 | 0.3803 | 0.0666 | 0.4916 | 0.0130 | 0.8931 |
| rs480807 | 0.1216 | 0.2085 | 0.0100 | 0.9181 | 0.0543 | 0.5744 |
| rs916430 | 0.1815 | 0.0591 | 0.0829 | 0.3894 | 0.0691 | 0.4724 |
| rs2359942 | 0.1062 | 0.2690 | 0.0850 | 0.3765 | 0.0137 | 0.8870 |
| rs717538 | 0.0995 | 0.3010 | 0.1026 | 0.2864 | 0.0342 | 0.7223 |
| rs2187942 | 0.0949 | 0.3236 | 0.1072 | 0.2655 | 0.0387 | 0.6872 |
| rs1417144 | 0.0838 | 0.3849 | 0.1541 | 0.1105 | 0.0793 | 0.4113 |
| rs647619 | 0.0288 | 0.7642 | 0.0743 | 0.4391 | 0.0152 | 0.8745 |
| rs1150903 | 0.0281 | 0.7702 | 0.0964 | 0.3160 | 0.0356 | 0.7106 |
| rs2501254 | 0.1524 | 0.1125 | 0.0008 | 0.9937 | 0.0463 | 0.6301 |
| rs953035 | 0.1353 | 0.1598 | 0.0365 | 0.7046 | 0.0228 | 0.8127 |
| rs677661 | 0.1208 | 0.2093 | 0.0281 | 0.7702 | 0.0038 | 0.9685 |
| rs595961 | 0.1457 | 0.1295 | 0.0046 | 0.9622 | 0.0683 | 0.4773 |
| rs272817 | 0.1352 | 0.1637 | 0.0892 | 0.3586 | 0.0783 | 0.4196 |
| rs4290027 | 0.1482 | 0.1252 | 0.0153 | 0.8744 | 0.1528 | 0.1139 |
| rs11211632 | 0.1930 | 0.0449 | 0.0076 | 0.9371 | 0.0745 | 0.4390 |
| rs12140284 | 0.0401 | 0.6845 | 0.0653 | 0.5084 | 0.0315 | 0.7499 |
| rs2793675 | 0.0409 | 0.6745 | 0.0873 | 0.3706 | 0.0008 | 0.9937 |
| rs11807256 | 0.0138 | 0.8867 | 0.2322 | 0.0169 | 0.0860 | 0.3755 |
| rs12565323 | 0.1257 | 0.1967 | 0.0749 | 0.4423 | 0.0062 | 0.9495 |
| rs1032429 | 0.2055 | 0.0324 | 0.0129 | 0.8932 | 0.0933 | 0.3315 |
| rs2275254 | 0.0796 | 0.4071 | 0.1055 | 0.2724 | 0.0493 | 0.6078 |
| rs2275603 | 0.1827 | 0.0580 | 0.1021 | 0.2897 | 0.0594 | 0.5377 |
| rs12042893 | 0.2273 | 0.0179 | 0.0849 | 0.3766 | 0.0803 | 0.4027 |
| rs10753700 | 0.0548 | 0.5695 | 0.1082 | 0.2620 | 0.0198 | 0.8373 |
| rs11581254 | 0.0170 | 0.8616 | 0.1579 | 0.1059 | 0.0286 | 0.7694 |
| rs7340104 | 0.0235 | 0.8067 | 0.0493 | 0.6078 | 0.0402 | 0.6757 |
| rs867426 | 0.1085 | 0.2589 | 0.0144 | 0.8807 | 0.0660 | 0.4921 |
| rs12145973 | 0.1963 | 0.0559 | 0.0190 | 0.8536 | 0.0422 | 0.6811 |
| rs12568035 | 0.1450 | 0.1362 | 0.0571 | 0.5575 | 0.0409 | 0.6744 |
| rs12034991 | 0.0877 | 0.3667 | 0.1216 | 0.2109 | 0.0954 | 0.3262 |
| rs9730058 | 0.1342 | 0.1623 | 0.0691 | 0.4725 | 0.1100 | 0.2523 |
| rs10910323 | 0.1760 | 0.0690 | 0.0046 | 0.9622 | 0.0428 | 0.6579 |
| rs716527 | 0.1459 | 0.1328 | 0.0231 | 0.8124 | 0.0461 | 0.6350 |
| rs12085924 | 0.0131 | 0.8929 | 0.1317 | 0.1757 | 0.0577 | 0.5526 |
| rs2484124 | 0.1981 | 0.0406 | 0.0191 | 0.8433 | 0.0727 | 0.4526 |
| rs548442 | 0.2064 | 0.0328 | 0.0092 | 0.9244 | 0.0872 | 0.3674 |
| rs640239 | 0.2239 | 0.0206 | 0.0268 | 0.7820 | 0.1016 | 0.2931 |
| rs10929675 | 0.0705 | 0.4628 | 0.0190 | 0.8435 | 0.0781 | 0.4161 |
| rs3732114 | 0.0713 | 0.4580 | 0.0213 | 0.8250 | 0.0728 | 0.4485 |
| rs6432127 | 0.0637 | 0.5072 | 0.0213 | 0.8250 | 0.0744 | 0.4391 |
| rs6733161 | 0.0394 | 0.6814 | 0.0440 | 0.6470 | 0.0940 | 0.3276 |
| rs11096686 | 0.2192 | 0.0225 | 0.0144 | 0.8808 | 0.0660 | 0.4922 |
| rs2193776 | 0.0077 | 0.9370 | 0.1411 | 0.1457 | 0.0889 | 0.3590 |
| rs4666014 | 0.1887 | 0.0501 | 0.0533 | 0.5803 | 0.0381 | 0.6929 |
| rs13006813 | 0.1168 | 0.2241 | 0.0137 | 0.8870 | 0.1259 | 0.1900 |
| rs12712519 | 0.1311 | 0.1720 | 0.0129 | 0.8932 | 0.1160 | 0.2271 |
| rs11689619 | 0.0887 | 0.3556 | 0.0296 | 0.7581 | 0.1039 | 0.2794 |
| rs341602 | 0.1163 | 0.2317 | 0.0208 | 0.8307 | 0.0501 | 0.6067 |
| rs4953260 | 0.1112 | 0.2487 | 0.0000 | 1.0000 | 0.1188 | 0.2177 |
| rs7591064 | 0.1284 | 0.1965 | 0.1117 | 0.2614 | 0.0112 | 0.9106 |
| rs12328023 | 0.1677 | 0.0822 | 0.0076 | 0.9370 | 0.1395 | 0.1483 |
| rs4671675 | 0.0903 | 0.3474 | 0.0767 | 0.4252 | 0.0948 | 0.3237 |
| rs6725950 | 0.0265 | 0.7823 | 0.0584 | 0.5432 | 0.0387 | 0.6872 |
| rs6546171 | 0.0825 | 0.3896 | 0.0887 | 0.3557 | 0.0871 | 0.3640 |
| rs2312547 | 0.1177 | 0.2210 | 0.0084 | 0.9308 | 0.0463 | 0.6300 |
| rs4852988 | 0.1406 | 0.1440 | 0.0540 | 0.5750 | 0.0327 | 0.7342 |
| rs17656058 | 0.1102 | 0.2622 | 0.0430 | 0.6618 | 0.0164 | 0.8674 |
| rs6752715 | 0.1962 | 0.0416 | 0.0061 | 0.9496 | 0.0837 | 0.3850 |
| rs942151 | 0.1222 | 0.2037 | 0.0266 | 0.7823 | 0.0478 | 0.6189 |
| rs7594352 | 0.1237 | 0.1981 | 0.0251 | 0.7944 | 0.0463 | 0.6301 |
| rs10496358 | 0.1598 | 0.1010 | 0.0749 | 0.4422 | 0.0347 | 0.7215 |
| rs6740690 | 0.1722 | 0.0752 | 0.0184 | 0.8495 | 0.0742 | 0.4431 |
| rs6743570 | 0.1722 | 0.0752 | 0.0184 | 0.8495 | 0.0742 | 0.4431 |
| rs1038511 | 0.0952 | 0.3234 | 0.0770 | 0.4249 | 0.0038 | 0.9685 |
| rs2683824 | 0.1318 | 0.1717 | 0.0313 | 0.7460 | 0.0442 | 0.6468 |
| rs2714174 | 0.1318 | 0.1717 | 0.0130 | 0.8932 | 0.0137 | 0.8869 |
| rs1402470 | 0.1314 | 0.1740 | 0.0046 | 0.9622 | 0.0535 | 0.5801 |
| rs13028997 | 0.1822 | 0.0581 | 0.0152 | 0.8745 | 0.0167 | 0.8621 |
| rs700550 | 0.1872 | 0.0520 | 0.0228 | 0.8127 | 0.0472 | 0.6243 |
| rs12053101 | 0.0283 | 0.7698 | 0.0606 | 0.5321 | 0.0345 | 0.7219 |
| rs17256082 | 0.0206 | 0.8311 | 0.0908 | 0.3470 | 0.0191 | 0.8434 |
| rs4549077 | 0.0784 | 0.4159 | 0.1013 | 0.2935 | 0.0038 | 0.9685 |
| rs13020779 | 0.1832 | 0.0570 | 0.0205 | 0.8311 | 0.0601 | 0.5327 |
| rs6718438 | 0.1708 | 0.0756 | 0.0418 | 0.6641 | 0.0448 | 0.6413 |
| rs6760896 | 0.2365 | 0.0138 | 0.0106 | 0.9120 | 0.1213 | 0.2065 |
| rs12612231 | 0.1276 | 0.1847 | 0.0122 | 0.8995 | 0.0258 | 0.7883 |
| rs3924658 | 0.0152 | 0.8745 | 0.1460 | 0.1294 | 0.0578 | 0.5484 |
| rs6709076 | 0.1277 | 0.1867 | 0.0130 | 0.8931 | 0.0512 | 0.5963 |
| rs2600306 | 0.0997 | 0.3009 | 0.0327 | 0.7341 | 0.0373 | 0.6988 |
| rs929707 | 0.0395 | 0.6814 | 0.1216 | 0.2064 | 0.0402 | 0.6756 |
| rs3732941 | 0.0015 | 0.9874 | 0.1476 | 0.1255 | 0.0319 | 0.7401 |
| rs1605527 | 0.0395 | 0.6814 | 0.0289 | 0.7641 | 0.0213 | 0.8250 |
| rs17008470 | 0.0750 | 0.4345 | 0.0174 | 0.8559 | 0.0235 | 0.8067 |
| rs4478120 | 0.0349 | 0.7165 | 0.0395 | 0.6814 | 0.0152 | 0.8745 |
| rs7642758 | 0.0311 | 0.7462 | 0.0402 | 0.6756 | 0.0144 | 0.8808 |
| rs9872812 | 0.0395 | 0.6813 | 0.0289 | 0.7641 | 0.0213 | 0.8250 |
| rs294278 | 0.0828 | 0.3894 | 0.0798 | 0.4070 | 0.0008 | 0.9937 |
| rs11710107 | 0.1370 | 0.1602 | 0.0596 | 0.5412 | 0.0023 | 0.9810 |
| rs2888312 | 0.1433 | 0.1421 | 0.0457 | 0.6396 | 0.0085 | 0.9304 |
| rs1986656 | 0.0222 | 0.8260 | 0.0230 | 0.8196 | 0.0485 | 0.6309 |
| rs2201057 | 0.0720 | 0.4573 | 0.0766 | 0.4291 | 0.0505 | 0.6018 |
| rs11710972 | 0.1615 | 0.0926 | 0.0266 | 0.7823 | 0.0402 | 0.6756 |
| rs6803290 | 0.2205 | 0.0227 | 0.0169 | 0.8618 | 0.0827 | 0.3929 |
| rs6770985 | 0.0281 | 0.7702 | 0.1207 | 0.2093 | 0.0023 | 0.9811 |
| rs9865950 | 0.1140 | 0.2451 | 0.0188 | 0.8485 | 0.0672 | 0.4936 |
| rs9866516 | 0.0728 | 0.4523 | 0.0115 | 0.9055 | 0.0115 | 0.9055 |
| rs9825550 | 0.1272 | 0.1962 | 0.0016 | 0.9873 | 0.0801 | 0.4158 |
| rs7614718 | 0.0501 | 0.6103 | 0.0078 | 0.9365 | 0.0595 | 0.5451 |
| rs2633604 | 0.2020 | 0.0357 | 0.0167 | 0.8621 | 0.1033 | 0.2829 |
| rs13082005 | 0.1443 | 0.1383 | 0.0425 | 0.6629 | 0.0887 | 0.3621 |
| rs10511316 | 0.0161 | 0.8681 | 0.0590 | 0.5426 | 0.0697 | 0.4718 |
| rs9851577 | 0.1045 | 0.2790 | 0.0176 | 0.8558 | 0.0099 | 0.9182 |
| rs4679354 | 0.0950 | 0.3236 | 0.0806 | 0.4025 | 0.0129 | 0.8932 |
| rs17282579 | 0.0925 | 0.3388 | 0.0850 | 0.3802 | 0.0467 | 0.6296 |
| rs7637370 | 0.0623 | 0.5173 | 0.0957 | 0.3197 | 0.0137 | 0.8870 |
| rs329134 | 0.0754 | 0.4342 | 0.0770 | 0.4249 | 0.0328 | 0.7341 |
| rs6439858 | 0.1729 | 0.0740 | 0.0107 | 0.9119 | 0.0291 | 0.7639 |
| rs9850224 | 0.0732 | 0.4520 | 0.0532 | 0.5849 | 0.0431 | 0.6575 |
| rs1399796 | 0.2043 | 0.0337 | 0.0433 | 0.6526 | 0.1025 | 0.2864 |
| rs9290505 | 0.1688 | 0.0795 | 0.0183 | 0.8496 | 0.0243 | 0.8005 |
| rs9855684 | 0.2192 | 0.0229 | 0.0320 | 0.7401 | 0.0928 | 0.3352 |
| rs7643541 | 0.1271 | 0.1871 | 0.0556 | 0.5642 | 0.0099 | 0.9182 |
| rs1719596 | 0.0150 | 0.8794 | 0.1288 | 0.1931 | 0.0813 | 0.4108 |
| rs9290933 | 0.1231 | 0.2008 | 0.0715 | 0.4579 | 0.0182 | 0.8497 |
| rs4677735 | 0.1528 | 0.1139 | 0.0275 | 0.7759 | 0.0642 | 0.5067 |
| rs7695691 | 0.0091 | 0.9245 | 0.0761 | 0.4296 | 0.0548 | 0.5696 |
| rs6599300 | 0.1279 | 0.1845 | 0.0335 | 0.7282 | 0.0426 | 0.6583 |
| rs3856982 | 0.1065 | 0.2689 | 0.0823 | 0.3936 | 0.0335 | 0.7282 |
| rs2063413 | 0.1025 | 0.2864 | 0.0433 | 0.6526 | 0.0570 | 0.5537 |
| rs6446675 | 0.0084 | 0.9307 | 0.0191 | 0.8433 | 0.0695 | 0.4719 |
| rs17713587 | 0.0466 | 0.6338 | 0.0591 | 0.5462 | 0.0124 | 0.8989 |
| rs4698374 | 0.0607 | 0.5276 | 0.0714 | 0.4579 | 0.0182 | 0.8497 |
| rs4698103 | 0.0835 | 0.3851 | 0.0311 | 0.7461 | 0.0531 | 0.5805 |
| rs4629443 | 0.0455 | 0.6357 | 0.0532 | 0.5804 | 0.0258 | 0.7883 |
| rs4575989 | 0.0433 | 0.6527 | 0.0555 | 0.5643 | 0.0220 | 0.8189 |
| rs10013604 | 0.1666 | 0.0837 | 0.0997 | 0.3007 | 0.0281 | 0.7701 |
| rs2345043 | 0.1512 | 0.1177 | 0.0138 | 0.8869 | 0.0107 | 0.9119 |
| rs1376307 | 0.1764 | 0.0679 | 0.0298 | 0.7579 | 0.0389 | 0.6869 |
| rs1155624 | 0.1049 | 0.2859 | 0.0689 | 0.4834 | 0.0861 | 0.3810 |
| rs1369093 | 0.1821 | 0.0581 | 0.0121 | 0.8995 | 0.0850 | 0.3765 |
| rs6828071 | 0.1420 | 0.1398 | 0.0494 | 0.6077 | 0.0190 | 0.8435 |
| rs11097328 | 0.1548 | 0.1073 | 0.0015 | 0.9874 | 0.0910 | 0.3434 |
| rs1019947 | 0.1100 | 0.2575 | 0.0423 | 0.6632 | 0.0285 | 0.7695 |
| rs1157180 | 0.1124 | 0.2476 | 0.0354 | 0.7156 | 0.0323 | 0.7394 |
| rs2659501 | 0.0357 | 0.7105 | 0.0814 | 0.3981 | 0.1338 | 0.1646 |
| rs1848116 | 0.0357 | 0.7105 | 0.0965 | 0.3159 | 0.1025 | 0.2864 |
| rs2850976 | 0.1681 | 0.0797 | 0.0348 | 0.7165 | 0.0250 | 0.7945 |
| rs2850350 | 0.1939 | 0.0433 | 0.0258 | 0.7884 | 0.0348 | 0.7165 |
| rs233992 | 0.1085 | 0.2589 | 0.0008 | 0.9937 | 0.0706 | 0.4628 |
| rs233990 | 0.0850 | 0.3765 | 0.0198 | 0.8373 | 0.0562 | 0.5590 |
| rs1016492 | 0.0782 | 0.4161 | 0.0084 | 0.9308 | 0.0622 | 0.5173 |
| rs7656744 | 0.0751 | 0.4344 | 0.0053 | 0.9559 | 0.0592 | 0.5380 |
| rs10857274 | 0.1269 | 0.1873 | 0.0783 | 0.4160 | 0.0038 | 0.9685 |
| rs1155422 | 0.1689 | 0.0806 | 0.0574 | 0.5532 | 0.0390 | 0.6868 |
| rs17315339 | 0.0592 | 0.5380 | 0.1246 | 0.1953 | 0.0213 | 0.8250 |
| rs10517729 | 0.1675 | 0.0884 | 0.0008 | 0.9936 | 0.1057 | 0.2824 |
| rs11934405 | 0.1355 | 0.1713 | 0.0722 | 0.4666 | 0.0452 | 0.6484 |
| rs6889488 | 0.1246 | 0.2065 | 0.0260 | 0.7919 | 0.0552 | 0.5757 |
| rs10520891 | 0.2018 | 0.0363 | 0.0023 | 0.9811 | 0.0700 | 0.4673 |
| rs11745765 | 0.1590 | 0.1001 | 0.0230 | 0.8125 | 0.0979 | 0.3115 |
| rs12186418 | 0.1267 | 0.1874 | 0.0023 | 0.9811 | 0.0129 | 0.8932 |
| rs13182372 | 0.1625 | 0.0924 | 0.0145 | 0.8807 | 0.0755 | 0.4341 |
| rs7736502 | 0.1606 | 0.0956 | 0.0114 | 0.9057 | 0.0693 | 0.4723 |
| rs7712010 | 0.1858 | 0.0539 | 0.0122 | 0.8994 | 0.0883 | 0.3595 |
| rs2289599 | 0.1476 | 0.1286 | 0.0138 | 0.8867 | 0.0154 | 0.8742 |
| rs5014235 | 0.1261 | 0.1943 | 0.0385 | 0.6923 | 0.0154 | 0.8742 |
| rs4342312 | 0.0182 | 0.8497 | 0.2295 | 0.0171 | 0.1230 | 0.2008 |
| rs2125714 | 0.0168 | 0.8619 | 0.0596 | 0.5375 | 0.0275 | 0.7760 |
| rs346860 | 0.1781 | 0.0655 | 0.0512 | 0.5964 | 0.0420 | 0.6638 |
| rs2546078 | 0.1638 | 0.0881 | 0.0015 | 0.9874 | 0.0910 | 0.3434 |
| rs29824 | 0.1480 | 0.1236 | 0.0691 | 0.4724 | 0.0736 | 0.4437 |
| rs13160298 | 0.0436 | 0.6522 | 0.0436 | 0.6522 | 0.0138 | 0.8868 |
| rs1316379 | 0.0055 | 0.9554 | 0.1380 | 0.1625 | 0.0307 | 0.7556 |
| rs9314015 | 0.0525 | 0.5857 | 0.0236 | 0.8065 | 0.0297 | 0.7580 |
| rs11135310 | 0.0487 | 0.6132 | 0.0365 | 0.7046 | 0.0395 | 0.6813 |
| rs4489081 | 0.0753 | 0.4343 | 0.0373 | 0.6987 | 0.0008 | 0.9937 |
| rs17376891 | 0.0467 | 0.6335 | 0.0522 | 0.5944 | 0.0451 | 0.6448 |
| rs11746351 | 0.0054 | 0.9558 | 0.0854 | 0.3796 | 0.1223 | 0.2082 |
| rs1500127 | 0.1335 | 0.1646 | 0.0592 | 0.5380 | 0.0182 | 0.8497 |
| rs565280 | 0.0951 | 0.3235 | 0.1241 | 0.1980 | 0.0175 | 0.8559 |
| rs1065154 | 0.0823 | 0.3936 | 0.1022 | 0.2898 | 0.0061 | 0.9496 |
| rs1650893 | 0.0823 | 0.3936 | 0.1083 | 0.2619 | 0.0061 | 0.9496 |
| rs11242715 | 0.1576 | 0.1034 | 0.1225 | 0.2058 | 0.0153 | 0.8743 |
| rs1138803 | 0.0516 | 0.5914 | 0.1017 | 0.2900 | 0.0804 | 0.4026 |
| rs2793434 | 0.0440 | 0.6470 | 0.0000 | 1.0000 | 0.0349 | 0.7164 |
| rs13340461 | 0.0927 | 0.3388 | 0.1050 | 0.2787 | 0.0391 | 0.6868 |
| rs4130023 | 0.1287 | 0.1840 | 0.0682 | 0.4815 | 0.0008 | 0.9937 |
| rs7746067 | 0.1832 | 0.0570 | 0.0251 | 0.7944 | 0.0707 | 0.4626 |
| rs9442831 | 0.1482 | 0.1236 | 0.0936 | 0.3313 | 0.0144 | 0.8807 |
| rs12111265 | 0.2300 | 0.0167 | 0.1147 | 0.2332 | 0.0615 | 0.5225 |
| rs7760979 | 0.1535 | 0.1107 | 0.0867 | 0.3680 | 0.0441 | 0.6470 |
| rs12202018 | 0.1462 | 0.1390 | 0.0040 | 0.9681 | 0.0324 | 0.7430 |
| rs9374007 | 0.1240 | 0.1980 | 0.0038 | 0.9685 | 0.0190 | 0.8435 |
| rs971403 | 0.1132 | 0.2419 | 0.0689 | 0.4767 | 0.0597 | 0.5374 |
| rs7755100 | 0.1117 | 0.2484 | 0.0391 | 0.6868 | 0.0321 | 0.7399 |
| rs7768952 | 0.1260 | 0.1944 | 0.0184 | 0.8494 | 0.0184 | 0.8494 |
| rs12198275 | 0.1275 | 0.1869 | 0.0390 | 0.6869 | 0.0466 | 0.6297 |
| rs9482421 | 0.1781 | 0.0636 | 0.0083 | 0.9308 | 0.1129 | 0.2394 |
| rs1322633 | 0.0876 | 0.3667 | 0.0600 | 0.5368 | 0.0100 | 0.9180 |
| rs9483335 | 0.0443 | 0.6467 | 0.0566 | 0.5586 | 0.0520 | 0.5910 |
| rs569833 | 0.1668 | 0.0869 | 0.0819 | 0.4008 | 0.0989 | 0.3103 |
| rs519332 | 0.1601 | 0.0971 | 0.0503 | 0.6021 | 0.0656 | 0.4969 |
| rs377962 | 0.1955 | 0.0460 | 0.0288 | 0.7687 | 0.0880 | 0.3691 |
| rs296422 | 0.1473 | 0.1256 | 0.0471 | 0.6244 | 0.0410 | 0.6698 |
| rs4896651 | 0.1042 | 0.2792 | 0.0419 | 0.6640 | 0.0190 | 0.8435 |
| rs11155550 | 0.2124 | 0.0271 | 0.0121 | 0.8995 | 0.0721 | 0.4532 |
| rs9397336 | 0.0733 | 0.4480 | 0.0550 | 0.5693 | 0.0596 | 0.5376 |
| rs4259254 | 0.0833 | 0.3920 | 0.1111 | 0.2537 | 0.0262 | 0.7876 |
| rs372454 | 0.1285 | 0.1820 | 0.0373 | 0.6988 | 0.0616 | 0.5224 |
| rs1517974 | 0.0334 | 0.7283 | 0.0942 | 0.3275 | 0.0714 | 0.4579 |
| rs2237315 | 0.1726 | 0.0719 | 0.0576 | 0.5485 | 0.0893 | 0.3516 |
| rs260368 | 0.0296 | 0.7582 | 0.1085 | 0.2589 | 0.0827 | 0.3895 |
| rs2345016 | 0.0379 | 0.6931 | 0.1076 | 0.2623 | 0.0681 | 0.4774 |
| rs17172715 | 0.0405 | 0.6752 | 0.0573 | 0.5532 | 0.0573 | 0.5533 |
| rs6593185 | 0.2038 | 0.0377 | 0.0367 | 0.7083 | 0.1148 | 0.2419 |
| rs2195139 | 0.1142 | 0.2432 | 0.1392 | 0.1553 | 0.0738 | 0.4507 |
| rs12534777 | 0.1180 | 0.2209 | 0.0084 | 0.9308 | 0.0274 | 0.7762 |
| rs7782875 | 0.2222 | 0.0207 | 0.0083 | 0.9308 | 0.1342 | 0.1623 |
| rs10254034 | 0.1050 | 0.2758 | 0.0183 | 0.8497 | 0.0145 | 0.8807 |
| rs12540386 | 0.0669 | 0.4871 | 0.0152 | 0.8745 | 0.0494 | 0.6077 |
| rs2077759 | 0.0911 | 0.3469 | 0.0329 | 0.7339 | 0.0145 | 0.8806 |
| rs13236941 | 0.0584 | 0.5432 | 0.0144 | 0.8807 | 0.0083 | 0.9308 |
| rs705308 | 0.2358 | 0.0149 | 0.0008 | 0.9937 | 0.0742 | 0.4431 |
| rs6465657 | 0.2313 | 0.0169 | 0.0107 | 0.9118 | 0.0728 | 0.4525 |
| rs11760238 | 0.0152 | 0.8745 | 0.0913 | 0.3433 | 0.0988 | 0.3046 |
| rs2188172 | 0.0000 | 1.0000 | 0.0760 | 0.4297 | 0.0760 | 0.4297 |
| rs13234705 | 0.0129 | 0.8932 | 0.0829 | 0.3893 | 0.0449 | 0.6413 |
| rs13235067 | 0.2024 | 0.0367 | 0.0621 | 0.5216 | 0.0529 | 0.5851 |
| rs2299546 | 0.0391 | 0.6866 | 0.1066 | 0.2714 | 0.0667 | 0.4913 |
| rs886001 | 0.0522 | 0.5906 | 0.1059 | 0.2749 | 0.0706 | 0.4667 |
| rs6957435 | 0.1049 | 0.2758 | 0.0700 | 0.4675 | 0.0973 | 0.3121 |
| rs7797420 | 0.1722 | 0.0731 | 0.0235 | 0.8066 | 0.1251 | 0.1927 |
| rs1922889 | 0.1166 | 0.2292 | 0.0261 | 0.7879 | 0.0552 | 0.5689 |
| rs2707575 | 0.1026 | 0.2892 | 0.0552 | 0.5690 | 0.0383 | 0.6925 |
| rs2189883 | 0.1062 | 0.2719 | 0.0742 | 0.4432 | 0.0038 | 0.9685 |
| rs993715 | 0.0491 | 0.6127 | 0.1028 | 0.2892 | 0.0476 | 0.6239 |
| rs2527049 | 0.1131 | 0.2444 | 0.0763 | 0.4330 | 0.0285 | 0.7695 |
| rs2623630 | 0.0638 | 0.5071 | 0.0365 | 0.7046 | 0.0835 | 0.3850 |
| rs2552241 | 0.0691 | 0.4724 | 0.0387 | 0.6872 | 0.0175 | 0.8559 |
| rs332040 | 0.2731 | 0.0046 | 0.1013 | 0.2934 | 0.1544 | 0.1088 |
| rs4077341 | 0.1637 | 0.0895 | 0.0328 | 0.7341 | 0.0617 | 0.5223 |
| rs11784487 | 0.1113 | 0.2487 | 0.0503 | 0.6020 | 0.0198 | 0.8372 |
| rs6472619 | 0.0349 | 0.7165 | 0.0379 | 0.6930 | 0.0500 | 0.6023 |
| rs4540401 | 0.0645 | 0.5022 | 0.0721 | 0.4532 | 0.0402 | 0.6756 |
| rs4425734 | 0.1554 | 0.1056 | 0.0083 | 0.9308 | 0.1160 | 0.2271 |
| rs2339654 | 0.0167 | 0.8621 | 0.1413 | 0.1419 | 0.0486 | 0.6133 |
| rs1463184 | 0.0235 | 0.8066 | 0.1481 | 0.1236 | 0.0402 | 0.6756 |
| rs2607061 | 0.0410 | 0.6698 | 0.1368 | 0.1552 | 0.0699 | 0.4676 |
| rs2045806 | 0.1475 | 0.1255 | 0.0091 | 0.9245 | 0.0259 | 0.7883 |
| rs1693569 | 0.0356 | 0.7106 | 0.1389 | 0.1485 | 0.0607 | 0.5276 |
| rs6981930 | 0.0464 | 0.6300 | 0.0981 | 0.3083 | 0.0753 | 0.4343 |
| rs4236799 | 0.1508 | 0.1178 | 0.0030 | 0.9748 | 0.0472 | 0.6243 |
| rs7817222 | 0.1478 | 0.1254 | 0.0030 | 0.9748 | 0.0442 | 0.6468 |
| rs4506180 | 0.0764 | 0.4292 | 0.0199 | 0.8371 | 0.0153 | 0.8744 |
| rs6996401 | 0.1440 | 0.1354 | 0.0191 | 0.8434 | 0.0891 | 0.3553 |
| rs7006331 | 0.1836 | 0.0561 | 0.0319 | 0.7402 | 0.0819 | 0.3938 |
| rs1876201 | 0.2339 | 0.0150 | 0.0684 | 0.4772 | 0.1002 | 0.2972 |
| rs2025324 | 0.0145 | 0.8807 | 0.1363 | 0.1574 | 0.0403 | 0.6755 |
| rs991250 | 0.1071 | 0.2655 | 0.0220 | 0.8188 | 0.0190 | 0.8435 |
| rs3923120 | 0.1386 | 0.1505 | 0.0488 | 0.6131 | 0.0061 | 0.9496 |
| rs1874108 | 0.0618 | 0.5221 | 0.1550 | 0.1087 | 0.0008 | 0.9937 |
| rs2811715 | 0.1752 | 0.0682 | 0.0083 | 0.9308 | 0.0857 | 0.3723 |
| rs13293826 | 0.1715 | 0.0774 | 0.0239 | 0.8061 | 0.0777 | 0.4239 |
| rs12554965 | 0.1313 | 0.1799 | 0.0117 | 0.9052 | 0.0148 | 0.8801 |
| rs11138465 | 0.2393 | 0.0143 | 0.0039 | 0.9684 | 0.0860 | 0.3786 |
| rs11793717 | 0.1591 | 0.0987 | 0.0328 | 0.7341 | 0.0145 | 0.8807 |
| rs11581 | 0.1161 | 0.2295 | 0.0245 | 0.8003 | 0.0451 | 0.6409 |
| rs12336938 | 0.0819 | 0.4007 | 0.0085 | 0.9305 | 0.0626 | 0.5208 |
| rs10512396 | 0.1641 | 0.0949 | 0.0031 | 0.9746 | 0.0352 | 0.7204 |
| rs1041205 | 0.1956 | 0.0417 | 0.0046 | 0.9622 | 0.0804 | 0.4027 |
| rs12552010 | 0.1865 | 0.0521 | 0.0349 | 0.7165 | 0.1274 | 0.1847 |
| rs13301412 | 0.1880 | 0.0502 | 0.0212 | 0.8250 | 0.0948 | 0.3237 |
| rs9423593 | 0.1414 | 0.1437 | 0.0512 | 0.5964 | 0.0023 | 0.9811 |
| rs12764801 | 0.1827 | 0.0580 | 0.0411 | 0.6697 | 0.0046 | 0.9622 |
| rs2398121 | 0.0534 | 0.5843 | 0.0969 | 0.3216 | 0.0441 | 0.6513 |
| rs7074891 | 0.0399 | 0.6847 | 0.1619 | 0.0993 | 0.0930 | 0.3434 |
| rs2795610 | 0.0918 | 0.3394 | 0.0266 | 0.7823 | 0.0281 | 0.7702 |
| rs7916768 | 0.1315 | 0.1739 | 0.0260 | 0.7881 | 0.0352 | 0.7161 |
| rs942576 | 0.1225 | 0.2060 | 0.0567 | 0.5586 | 0.0567 | 0.5586 |
| rs7076053 | 0.1915 | 0.0473 | 0.0206 | 0.8310 | 0.0954 | 0.3232 |
| rs10762485 | 0.1100 | 0.2523 | 0.1009 | 0.2937 | 0.1024 | 0.2865 |
| rs10786777 | 0.1568 | 0.1037 | 0.0594 | 0.5378 | 0.0487 | 0.6132 |
| rs822094 | 0.1422 | 0.1397 | 0.0114 | 0.9057 | 0.0030 | 0.9748 |
| rs1570269 | 0.0791 | 0.4114 | 0.0381 | 0.6929 | 0.0365 | 0.7046 |
| rs1431486 | 0.0618 | 0.5222 | 0.1686 | 0.0808 | 0.0785 | 0.4158 |
| rs1931725 | 0.1966 | 0.0409 | 0.1101 | 0.2522 | 0.0918 | 0.3394 |
| rs12415467 | 0.0818 | 0.3977 | 0.0681 | 0.4817 | 0.0176 | 0.8557 |
| rs4751118 | 0.1872 | 0.0520 | 0.0503 | 0.6021 | 0.0944 | 0.3274 |
| rs3123171 | 0.1940 | 0.0447 | 0.0367 | 0.7044 | 0.0527 | 0.5855 |
| rs11601498 | 0.0958 | 0.3390 | 0.0341 | 0.7336 | 0.0122 | 0.9033 |
| rs12804437 | 0.0942 | 0.3473 | 0.0357 | 0.7214 | 0.0138 | 0.8904 |
| rs7935223 | 0.1124 | 0.2539 | 0.1085 | 0.2709 | 0.0299 | 0.7617 |
| rs12576777 | 0.1241 | 0.1979 | 0.0236 | 0.8065 | 0.0114 | 0.9057 |
| rs2196961 | 0.1449 | 0.1333 | 0.0015 | 0.9874 | 0.0336 | 0.7281 |
| rs7927476 | 0.1285 | 0.1820 | 0.0555 | 0.5643 | 0.1026 | 0.2864 |
| rs1346690 | 0.0684 | 0.4772 | 0.0883 | 0.3596 | 0.0548 | 0.5696 |
| rs7121400 | 0.0738 | 0.4437 | 0.0723 | 0.4531 | 0.0434 | 0.6526 |
| rs708228 | 0.1432 | 0.1408 | 0.0909 | 0.3501 | 0.0693 | 0.4760 |
| rs7930102 | 0.1978 | 0.0419 | 0.0547 | 0.5741 | 0.1054 | 0.2782 |
| rs6591555 | 0.0342 | 0.7224 | 0.0722 | 0.4532 | 0.0144 | 0.8807 |
| rs7129085 | 0.0015 | 0.9874 | 0.0671 | 0.4869 | 0.0853 | 0.3763 |
| rs2282529 | 0.1483 | 0.1295 | 0.0179 | 0.8551 | 0.0675 | 0.4898 |
| rs1135029 | 0.0894 | 0.3551 | 0.1109 | 0.2517 | 0.0344 | 0.7221 |
| rs341087 | 0.0463 | 0.6300 | 0.0342 | 0.7223 | 0.0030 | 0.9748 |
| rs10793036 | 0.0427 | 0.6581 | 0.1359 | 0.1595 | 0.0168 | 0.8620 |
| rs11235559 | 0.0882 | 0.3596 | 0.0776 | 0.4205 | 0.0228 | 0.8127 |
| rs189332 | 0.1013 | 0.2935 | 0.0510 | 0.5967 | 0.0236 | 0.8066 |
| rs10898866 | 0.1827 | 0.0589 | 0.0015 | 0.9874 | 0.0971 | 0.3155 |
| rs7107376 | 0.0316 | 0.7453 | 0.0601 | 0.5365 | 0.0516 | 0.5955 |
| rs11603266 | 0.1502 | 0.1196 | 0.0145 | 0.8807 | 0.0862 | 0.3719 |
| rs7940652 | 0.1352 | 0.1671 | 0.0296 | 0.7629 | 0.0187 | 0.8489 |
| rs10891727 | 0.1521 | 0.1235 | 0.1514 | 0.1254 | 0.0441 | 0.6549 |
| rs431736 | 0.2412 | 0.0123 | 0.0343 | 0.7223 | 0.1027 | 0.2863 |
| rs3809254 | 0.1092 | 0.2556 | 0.0061 | 0.9496 | 0.0030 | 0.9748 |
| rs10849446 | 0.1295 | 0.1793 | 0.0000 | 1.0000 | 0.0289 | 0.7640 |
| rs3759324 | 0.0790 | 0.4115 | 0.0441 | 0.6469 | 0.0091 | 0.9245 |
| rs2192973 | 0.2064 | 0.0329 | 0.0061 | 0.9496 | 0.0336 | 0.7280 |
| rs10840658 | 0.1682 | 0.0832 | 0.0038 | 0.9684 | 0.0407 | 0.6750 |
| rs2016977 | 0.0644 | 0.5185 | 0.1103 | 0.2688 | 0.0121 | 0.9036 |
| rs3782525 | 0.0644 | 0.5185 | 0.1103 | 0.2688 | 0.0121 | 0.9036 |
| rs939856 | 0.1788 | 0.0635 | 0.0038 | 0.9685 | 0.0799 | 0.4069 |
| rs7305924 | 0.1002 | 0.2973 | 0.0258 | 0.7883 | 0.0486 | 0.6133 |
| rs10771234 | 0.1680 | 0.0810 | 0.0403 | 0.6755 | 0.0494 | 0.6077 |
| rs1011870 | 0.1918 | 0.0465 | 0.0305 | 0.7520 | 0.0282 | 0.7701 |
| rs7977896 | 0.1955 | 0.0424 | 0.0373 | 0.6987 | 0.0320 | 0.7401 |
| rs12828155 | 0.2244 | 0.0202 | 0.0229 | 0.8126 | 0.0771 | 0.4248 |
| rs1909340 | 0.0895 | 0.3516 | 0.0690 | 0.4725 | 0.0076 | 0.9371 |
| rs3741604 | 0.1462 | 0.1293 | 0.0213 | 0.8249 | 0.0640 | 0.5070 |
| rs7954956 | 0.1090 | 0.2586 | 0.0282 | 0.7701 | 0.0373 | 0.6987 |
| rs12371477 | 0.1323 | 0.1693 | 0.0335 | 0.7282 | 0.0532 | 0.5803 |
| rs11110445 | 0.0990 | 0.3044 | 0.0335 | 0.7281 | 0.0381 | 0.6929 |
| rs10861946 | 0.0661 | 0.4921 | 0.0631 | 0.5122 | 0.0601 | 0.5327 |
| rs2541886 | 0.1746 | 0.0704 | 0.0099 | 0.9182 | 0.0259 | 0.7882 |
| rs482522 | 0.2399 | 0.0128 | 0.0549 | 0.5695 | 0.0716 | 0.4577 |
| rs558275 | 0.2636 | 0.0063 | 0.0648 | 0.5019 | 0.0891 | 0.3553 |
| rs1859455 | 0.1485 | 0.1235 | 0.0221 | 0.8188 | 0.0647 | 0.5019 |
| rs2615666 | 0.2222 | 0.0211 | 0.0289 | 0.7640 | 0.0868 | 0.3679 |
| rs1859458 | 0.1738 | 0.0717 | 0.0549 | 0.5694 | 0.0518 | 0.5911 |
| rs2346115 | 0.0015 | 0.9874 | 0.0546 | 0.5742 | 0.0707 | 0.4666 |
| rs2146136 | 0.1153 | 0.2328 | 0.0848 | 0.3804 | 0.0283 | 0.7700 |
| rs4943130 | 0.0876 | 0.3636 | 0.0236 | 0.8065 | 0.0632 | 0.5120 |
| rs9563673 | 0.1165 | 0.2268 | 0.0053 | 0.9559 | 0.0480 | 0.6187 |
| rs9565069 | 0.1216 | 0.2064 | 0.0806 | 0.4025 | 0.0851 | 0.3764 |
| rs1014936 | 0.1603 | 0.0957 | 0.0509 | 0.5967 | 0.0479 | 0.6188 |
| rs9543335 | 0.1829 | 0.0579 | 0.0145 | 0.8807 | 0.0015 | 0.9874 |
| rs9543336 | 0.1829 | 0.0579 | 0.0145 | 0.8807 | 0.0015 | 0.9874 |
| rs9300574 | 0.1470 | 0.1317 | 0.0782 | 0.4229 | 0.0642 | 0.5101 |
| rs1890139 | 0.1015 | 0.2933 | 0.0053 | 0.9559 | 0.0160 | 0.8682 |
| rs1958313 | 0.0457 | 0.6354 | 0.0839 | 0.3847 | 0.0030 | 0.9748 |
| rs225843 | 0.0934 | 0.3315 | 0.0706 | 0.4627 | 0.0539 | 0.5751 |
| rs1402517 | 0.1620 | 0.0950 | 0.0069 | 0.9432 | 0.0806 | 0.4060 |
| rs1057804 | 0.1691 | 0.0783 | 0.0008 | 0.9937 | 0.1426 | 0.1377 |
| rs2057367 | 0.1454 | 0.1329 | 0.0168 | 0.8618 | 0.0398 | 0.6808 |
| rs1957358 | 0.1854 | 0.0540 | 0.0426 | 0.6583 | 0.0654 | 0.4970 |
| rs1289407 | 0.0590 | 0.5425 | 0.1112 | 0.2514 | 0.0253 | 0.7941 |
| rs7151279 | 0.0956 | 0.3198 | 0.0304 | 0.7521 | 0.1032 | 0.2829 |
| rs2594934 | 0.1820 | 0.0581 | 0.0137 | 0.8870 | 0.0728 | 0.4485 |
| rs2594935 | 0.1688 | 0.0796 | 0.0228 | 0.8127 | 0.0768 | 0.4251 |
| rs11071503 | 0.1563 | 0.1038 | 0.0152 | 0.8745 | 0.0387 | 0.6872 |
| rs11854089 | 0.1344 | 0.1725 | 0.0354 | 0.7196 | 0.0542 | 0.5820 |
| rs8023445 | 0.0106 | 0.9120 | 0.1898 | 0.0484 | 0.0592 | 0.5380 |
| rs1011051 | 0.2170 | 0.0244 | 0.0450 | 0.6412 | 0.0906 | 0.3472 |
| rs2278295 | 0.2214 | 0.0215 | 0.0343 | 0.7223 | 0.0921 | 0.3392 |
| rs4332691 | 0.2343 | 0.0158 | 0.0231 | 0.8123 | 0.1137 | 0.2415 |
| rs7175118 | 0.1582 | 0.1017 | 0.0329 | 0.7339 | 0.0588 | 0.5427 |
| rs566160 | 0.1821 | 0.0606 | 0.0854 | 0.3795 | 0.1268 | 0.1914 |
| rs341399 | 0.2239 | 0.0198 | 0.0190 | 0.8435 | 0.0296 | 0.7581 |
| rs4776758 | 0.0498 | 0.6072 | 0.0821 | 0.3974 | 0.0008 | 0.9937 |
| rs4776759 | 0.0991 | 0.3044 | 0.0885 | 0.3595 | 0.0434 | 0.6525 |
| rs11071869 | 0.0992 | 0.3044 | 0.0855 | 0.3762 | 0.0481 | 0.6187 |
| rs10518680 | 0.1229 | 0.2033 | 0.0237 | 0.8065 | 0.0191 | 0.8434 |
| rs10518938 | 0.0637 | 0.5072 | 0.0501 | 0.6023 | 0.0334 | 0.7283 |
| rs980618 | 0.1961 | 0.0416 | 0.0289 | 0.7641 | 0.0198 | 0.8373 |
| rs1896799 | 0.0175 | 0.8559 | 0.0814 | 0.3981 | 0.0874 | 0.3638 |
| rs3825877 | 0.0342 | 0.7223 | 0.0281 | 0.7701 | 0.0190 | 0.8435 |
| rs2292463 | 0.0342 | 0.7223 | 0.0281 | 0.7701 | 0.0190 | 0.8435 |
| rs1107179 | 0.0228 | 0.8127 | 0.0563 | 0.5589 | 0.0373 | 0.6988 |
| rs4287543 | 0.2033 | 0.0343 | 0.0410 | 0.6698 | 0.1244 | 0.1953 |
| rs4932557 | 0.1898 | 0.0484 | 0.0167 | 0.8621 | 0.1427 | 0.1377 |
| rs4632107 | 0.1941 | 0.0440 | 0.0587 | 0.5430 | 0.1013 | 0.2934 |
| rs7495265 | 0.1708 | 0.0756 | 0.0311 | 0.7461 | 0.0918 | 0.3394 |
| rs4932583 | 0.1696 | 0.0782 | 0.0160 | 0.8683 | 0.0053 | 0.9559 |
| rs4777882 | 0.1616 | 0.0964 | 0.0693 | 0.4761 | 0.1062 | 0.2745 |
| rs1011489 | 0.2346 | 0.0150 | 0.0351 | 0.7163 | 0.1142 | 0.2361 |
| rs4786739 | 0.1881 | 0.0510 | 0.0084 | 0.9307 | 0.0754 | 0.4341 |
| rs12929785 | 0.1570 | 0.1062 | 0.0223 | 0.8184 | 0.0346 | 0.7216 |
| rs7201128 | 0.2205 | 0.0224 | 0.0237 | 0.8065 | 0.1045 | 0.2789 |
| rs9989425 | 0.3260 | 0.0008 | 0.1210 | 0.2117 | 0.1500 | 0.1213 |
| rs2369535 | 0.1952 | 0.0424 | 0.0144 | 0.8807 | 0.0843 | 0.3807 |
| rs756802 | 0.1679 | 0.0810 | 0.0144 | 0.8807 | 0.0646 | 0.5021 |
| rs4889551 | 0.1414 | 0.1543 | 0.0493 | 0.6198 | 0.0024 | 0.9808 |
| rs933561 | 0.0951 | 0.3266 | 0.0384 | 0.6924 | 0.0199 | 0.8370 |
| rs806739 | 0.0788 | 0.4116 | 0.0819 | 0.3938 | 0.0061 | 0.9496 |
| rs2550904 | 0.1475 | 0.1255 | 0.0183 | 0.8497 | 0.0608 | 0.5275 |
| rs3784929 | 0.1625 | 0.0911 | 0.0213 | 0.8250 | 0.0349 | 0.7164 |
| rs9929377 | 0.1050 | 0.2975 | 0.0755 | 0.4539 | 0.0205 | 0.8387 |
| rs2580312 | 0.2197 | 0.0229 | 0.0153 | 0.8744 | 0.1404 | 0.1460 |
| rs4888116 | 0.2023 | 0.0357 | 0.0411 | 0.6698 | 0.0517 | 0.5912 |
| rs16957806 | 0.2660 | 0.0057 | 0.0700 | 0.4675 | 0.1322 | 0.1694 |
| rs2966849 | 0.1398 | 0.1462 | 0.0122 | 0.8994 | 0.0023 | 0.9811 |
| rs3785415 | 0.2623 | 0.0064 | 0.0601 | 0.5327 | 0.0965 | 0.3159 |
| rs9908079 | 0.2361 | 0.0151 | 0.0900 | 0.3544 | 0.0715 | 0.4617 |
| rs10521211 | 0.1284 | 0.1820 | 0.0859 | 0.3722 | 0.0327 | 0.7342 |
| rs2525570 | 0.1373 | 0.1530 | 0.0554 | 0.5644 | 0.0736 | 0.4438 |
| rs1055636 | 0.1874 | 0.0551 | 0.0171 | 0.8615 | 0.0124 | 0.8991 |
| rs11079764 | 0.1953 | 0.0431 | 0.0244 | 0.8004 | 0.0275 | 0.7761 |
| rs10491196 | 0.1011 | 0.2936 | 0.0327 | 0.7342 | 0.0426 | 0.6583 |
| rs6505045 | 0.0907 | 0.3472 | 0.0252 | 0.7943 | 0.0145 | 0.8807 |
| rs9652853 | 0.1440 | 0.1354 | 0.0358 | 0.7104 | 0.0053 | 0.9559 |
| rs12940715 | 0.0594 | 0.5560 | 0.0727 | 0.4717 | 0.0066 | 0.9478 |
| rs1071664 | 0.2204 | 0.0228 | 0.0398 | 0.6809 | 0.0184 | 0.8495 |
| rs11150843 | 0.0732 | 0.4482 | 0.0061 | 0.9496 | 0.0671 | 0.4869 |
| rs168206 | 0.1123 | 0.2426 | 0.0121 | 0.8995 | 0.0197 | 0.8373 |
| rs11662508 | 0.1873 | 0.0512 | 0.0266 | 0.7823 | 0.0538 | 0.5751 |
| rs4800158 | 0.1193 | 0.2150 | 0.0373 | 0.6988 | 0.0372 | 0.6988 |
| rs4800476 | 0.1268 | 0.1873 | 0.0524 | 0.5859 | 0.0387 | 0.6872 |
| rs2047683 | 0.0615 | 0.5224 | 0.0889 | 0.3555 | 0.0099 | 0.9182 |
| rs356874 | 0.0030 | 0.9748 | 0.1273 | 0.1871 | 0.0975 | 0.3119 |
| rs2276209 | 0.0418 | 0.6641 | 0.0996 | 0.3009 | 0.0600 | 0.5327 |
| rs10401065 | 0.1077 | 0.2623 | 0.2155 | 0.0249 | 0.1092 | 0.2556 |
| rs9946933 | 0.1880 | 0.0502 | 0.0106 | 0.9120 | 0.0531 | 0.5805 |
| rs12604430 | 0.0895 | 0.3516 | 0.0076 | 0.9371 | 0.0349 | 0.7165 |
| rs9955285 | 0.2231 | 0.0203 | 0.0213 | 0.8250 | 0.0698 | 0.4676 |
| rs6507652 | 0.2200 | 0.0220 | 0.0182 | 0.8497 | 0.0698 | 0.4676 |
| rs4622605 | 0.1680 | 0.0832 | 0.0253 | 0.7940 | 0.1097 | 0.2579 |
| rs11665332 | 0.1573 | 0.1035 | 0.1077 | 0.2651 | 0.0366 | 0.7044 |
| rs10409243 | 0.0799 | 0.4068 | 0.0815 | 0.3979 | 0.0084 | 0.9308 |
| rs1363347 | 0.1427 | 0.1442 | 0.0590 | 0.5463 | 0.0527 | 0.5894 |
| rs4805985 | 0.1176 | 0.2210 | 0.0509 | 0.5968 | 0.0698 | 0.4676 |
| rs7259736 | 0.1620 | 0.0925 | 0.0343 | 0.7223 | 0.0251 | 0.7944 |
| rs8112457 | 0.1502 | 0.1196 | 0.0656 | 0.4968 | 0.0023 | 0.9811 |
| rs989251 | 0.1556 | 0.1070 | 0.0618 | 0.5222 | 0.0015 | 0.9874 |
| rs2290652 | 0.2670 | 0.0057 | 0.0260 | 0.7882 | 0.1236 | 0.2005 |
| rs7253017 | 0.1604 | 0.0970 | 0.0558 | 0.5639 | 0.0031 | 0.9748 |
| rs873711 | 0.1268 | 0.1873 | 0.0327 | 0.7342 | 0.0676 | 0.4822 |
| rs677344 | 0.0260 | 0.7880 | 0.0866 | 0.3714 | 0.0168 | 0.8618 |
| rs4814097 | 0.1662 | 0.0838 | 0.0311 | 0.7461 | 0.0751 | 0.4344 |
| rs2327960 | 0.1342 | 0.1623 | 0.0357 | 0.7106 | 0.0584 | 0.5432 |
| rs331617 | 0.0282 | 0.7700 | 0.0634 | 0.5119 | 0.0527 | 0.5856 |
| rs6026302 | 0.1231 | 0.2008 | 0.0167 | 0.8621 | 0.0410 | 0.6698 |
| rs2825726 | 0.1748 | 0.0723 | 0.0455 | 0.6404 | 0.0655 | 0.5010 |
| rs2825730 | 0.1951 | 0.0431 | 0.0519 | 0.5910 | 0.0610 | 0.5273 |
| rs7287616 | 0.0729 | 0.4689 | 0.0590 | 0.5579 | 0.0737 | 0.4639 |
| rs5752178 | 0.1481 | 0.1236 | 0.0160 | 0.8683 | 0.1344 | 0.1622 |
| rs4386 | 0.2180 | 0.0234 | 0.0023 | 0.9811 | 0.1527 | 0.1125 |
| rs1894599 | 0.1491 | 0.1216 | 0.1172 | 0.2239 | 0.0715 | 0.4579 |
| rs8142159 | 0.1634 | 0.0895 | 0.0479 | 0.6188 | 0.0456 | 0.6356 |
| rs7287117 | 0.1769 | 0.0696 | 0.0209 | 0.8306 | 0.0564 | 0.5630 |
| rs5937220 | 0.1700 | 0.0781 | 0.0053 | 0.9559 | 0.0099 | 0.9182 |
| rs5917027 | 0.1189 | 0.2262 | 0.0305 | 0.7562 | 0.0156 | 0.8735 |
