# Supplementary material for: Genome-Wide Identification of Susceptibility Alleles for Viral Infections through a Population Genetics Approach
Source: PLoS Genet. 2010 Feb 19;6(2):e1000849. doi: 10.1371/journal.pgen.1000849 (PMC2824813; doi:10.1371/journal.pgen.1000849)
Supplement: Table S6 — List of viruses identified in at least one country (n = 81) (0.01 MB DOC) [file pgen.1000849.s006.doc]

Table S6.

| List of viruses identified in at least one country (n=81) |
| --- |
| Banna virus, Sabia virus, Orthobunyavirus, California encephalitis group viruses, Chandipura virus, Chikingunya virus, Cowpox virus, CCHF virus, Dengue virus, Eastern equine encephalitis virus, Ebola virus, Group C fever viruses, Hantavirus (Hantaan, Puumala, Dobrava/Belgrade, Saaremaa and Seoul viruses), Hantavirus-pulmonary syndrome (Sin Nombre, Black Creek Canal, Bayou, New York-1, Andes), Hendra virus, Ilheus virus and Bussuquara virus, Japanese encephalitis virus, Lassa virus, Marburg virus, Mayaro virus, Monkeypox virus, New world Phleboviruses (Alenquer, Arboledas, Bujaru, Cacao, Candiru, Chagres and Punta Toro viruses), O'nyong nyong virus, Omsk hemorrhagic fever virus, Powassan virus, Rift Valley fever virus, Rocio virus, Ross River virus, Sandfly fever virus, Sindbis virus, Spondweni virus, St. Louis encephalitis virus, Tanapox virus, Thogoto virus, Central European encephalitis virus, Venezuelan equine encephalitis virus, Wesselsbron virus, West nile virus, Western equine encephalitis virus, Yellow fever virus, Zika virus, Louping ill virus, Murray valley encephalitis virus, Nipah virus, Oropouche virus, Russian spring-summer virus, Chandipura virus, Vesicular stomatitis virus, Adenovirus, HIV, Rhinoviruses, Coronavirus, Cytomegalovirus, Picornaviridae (Coxsackievirus, ECHO virus, Enterovirus, Parechovirus), Calicivirus (Norwalk, Hawaii, Sapporo, Snow Mountain, Norovirus), Hepatitis A virus, Hepatitis B virus, Hepatitis C virus, Hepatitis E virus, Hepatitis D virus, Hepatitis G virus, Herpes virus I, Herpes virus II , Herpes zoster, EBV, Influenza virus, Parainfluenza virus, Measles virus, Mumps virus, Erythrovirus B19, Polio virus, Pseudocowpox virus, Rabies virus, Human respiratory syncytial virus, Human Metapneumovirus, Roseolovirus, Rotavirus, Rubella virus, Variola virus, Lymphocytic choriomeningitis virus, Orf virus |
